# Identification of Structural and Morphogenesis Genes of *Sulfitobacter* Phage ΦGT1 and Placement within the Evolutionary History of the Podoviruses

**DOI:** 10.3390/v15071475

**Published:** 2023-06-29

**Authors:** Stephen C. Hardies, Byung Cheol Cho, Gwang Il Jang, Zhiqing Wang, Chung Yeon Hwang

**Affiliations:** 1 Department of Biochemistry and Structural Biology, UT Health, San Antonio, TX 78229, USA; 2 Microbial Oceanography Laboratory, School of Earth and Environmental Sciences and Research Institute of Oceanography, Seoul National University, Seoul 08826, Republic of Korea; bccho@snu.ac.kr; 3 Saemangeum Environmental Research Center, Kunsan National University, Gunsan 54150, Republic of Korea; 4 Aquatic Disease Control Division, National Fishery Products Quality Management Service, Busan 46083, Republic of Korea; gijang2@korea.kr; 5 National Cryo-EM Facility, Cancer Research Technology Program, Frederick National Laboratory for Cancer Research, Leidos Biomedical Research Inc., Frederick, MD 21702, USA; zhiqing.wang@nih.gov

**Keywords:** marine virus, podovirus, tubular tail A protein, timetree, bacteriophage evolution

## Abstract

ΦGT1 is a lytic podovirus of an alphaproteobacterial *Sulfitobacter* species, with few closely matching sequences among characterized phages, thus defying a useful description by simple sequence clustering methods. The history of the ΦGT1 core structure module was reconstructed using timetrees, including numerous related prospective prophages, to flesh out the evolutionary lineages spanning from the origin of the ejectosomal podovirus >3.2 Gya to the present genes of ΦGT1 and its closest relatives. A peculiarity of the ΦGT1 structural proteome is that it contains two paralogous tubular tail A (tubeA) proteins. The origin of the dual tubeA arrangement was traced to a recombination between two more ancient podoviral lineages occurring ~0.7 Gya in the alphaproteobacterial order *Rhizobiales*. Descendants of the ancestral dual A recombinant were tracked forward forming both temperate and lytic phage clusters and exhibiting both vertical transmission with patchy persistence and horizontal transfer with respect to host taxonomy. The two ancestral lineages were traced backward, making junctions with a major metagenomic podoviral family, the LUZ24-like gammaproteobacterial phages, and Myxococcal phage Mx8, and finally joining near the origin of podoviruses with P22. With these most conservative among phage genes, deviations from uncomplicated vertical and nonrecombinant descent are numerous but countable. The use of timetrees allowed conceptualization of the phage’s evolution in the context of a sequence of ancestors spanning the time of life on Earth.

## 1. Introduction

ΦGT1 is a lytic podovirus of *Sulfitobacter*. Both the host and phage were isolated from a tidal flat and sequenced [1]. *Sulfitobacter* is a genus in the marine family *Roseobacteraceae* of the order *Rhodobacterales* of alphaproteobacteria [2]. There are relatively few characterized phages of cultured alphaproteobacteria despite metagenomics data indicating that phages of alphaproteobacteria are highly abundant in the world’s oceans [3]. Observations from Hwang et al. [1] and its associated GenBank file (MT584811) include that the closest Blast matches are to three temperate podoviruses of *Sulfitobacter marinus* [4], including similarity in frame organization of both structural and nonstructural gene modules. However, the level of similarity in the structural genes is much stronger. The large terminase belongs to pfam04466. Pfam04466 is a subset of the terminases called P22-like [5]. The tubular tail B (tubeB) protein is distantly P22-like. In addition, there is a weak similarity to P22 ejection proteins (analogous to T7 internal virion proteins). These observations suggest that P22 may be a useful prototype for further comparative studies. However, the P22 matches are far away, requiring profile or Hidden Markov Matrix (HMM) methodology for detection.

Sequenced phages that match by BlastP or Psi-Blast more closely than P22 and which we will place into phylogenetic context are as follows. They are a mixture of lytic and temperate phages and are scattered across a broad host taxonomic range. For most structural genes, the next closest match beyond the *Sulfitobacter* phages is EPV2, also known as EBPR2 [6]. This is a metagenomic construct from a paper mostly about EPV1, which is from a betaproteobacterial host. However, the closest Blast matches of the nonstructural genes of EPV2 (including an integrase) are mostly found in chromosomes of *Roseobacteraceae* or other alphaproteobacteria, not betaproteobacteria. So EPV2 probably represents an alphaproteobacterial phage of *Roseobacteraceae* or an ancestral bacterium thereof. EPV2 is the only other currently sequenced representative of the ΦGT1 lineage after its entry into *Rhodobacterales*, apparently by ~0.35 Gya. Beyond EPV2 there are Blast matches to the lytic *Brucella* phages [7], also known as Perisiviruses with prototype phage Pr; or to temperate *Sinorhizobium* phage ΦM5 [8]. These both inhabit *Rhizobiales* (aka. *Hypomicrobiales*), which is a different alphaproteobacterial order than the one in which ΦGT1 itself resides. More distant matches are found to a large collection of lytic gammaproteobacterial phages, including *Pseudomonas* phage LUZ24 [9], Enterobacteria phage phiEco32 [10], *Salmonella* phage 7–11 [11], and *Vibrio* phage VPp1 [12], or to temperate deltaproteobacterial phage Mx8 [13], and temperate phage 45A6 of *Rhodobacterales* [14]. The relationship of ΦM5 to LUZ24 has been previously noted [8]. Only virion structural genes exhibit this matching pattern, and those that match ΦM5 and the gammaproteobacterial group do not match the Mx8, *Brucella* phage, 45A6 cluster, and vice versa. To complement this matching pattern of few and distant matches, sequences from numerous prospective prophages from the expanding library of sequenced bacterial genomes were used. Unlike the sparse representation of the ΦGT1 lineage in *Rhodobacterales*, there appears to have been vigorous propagation of ΦGT1-like temperate phages in *Rhizobiales*.

The most common way of conducting comparative characterization of phages is to cluster them by the number of matches found by BlastP or other similarity detection programs. The International Committee on Taxonomy of Viruses (ICTV) publishes a formal taxonomy mainly based on this concept. The prior concept of clustering by tail morphotypes into families [15]—in this case, podoviruses—is currently being discarded by ICTV [16]. The Blast-based methodology is unable to deal with highly divergent relationships (such as the relationship of ΦGT1 to the closest well-characterized prototype, P22). The disparate phages mentioned above were all previously classified in *Podoviridae*, but are now either unclassified or scattered into numerous small disjoint taxa. ICTV has been proposing alternative family names for some clusters of lytic phages. The process of building a phage taxonomy tends to be confounded by recombinational gene reassortment among phages over time. A recent study [17] demonstrated that temperate phages exhibit about 10-fold more recombinational reassortment than purely lytic phage families. Correspondingly, the coalescing of temperate phages into new ICTV families has been slow compared to lytic phages. Of the phages mentioned above, the gammaproteobacterial phages are a lytic group that will probably eventually qualify as an ICTV family. It is currently splintered into *Bruynogheviruses*, *Krylovviruses*, *Bjornviruses*, *Vicosaviruses*, and *Kuraviruses* (at least). We will show that the pathway linking ΦGT1 to the gammaproteobacterial group is through a large population of recombinogenic alphaproteobacterial temperate phages, known only through bacterial genome sequencing and unlikely to appear in the ICTV hierarchy any time soon.

We approached this problem through timetrees rather than a clustering approach. The deepest homology relationships that could be detected with HMM comparisons were organized into time-scaled phylogenetic gene trees for those genes that were amenable. The scaling was founded on a global large terminase subunit alignment [18] which produces a timetree scaled by rooting at the origin of life on Earth [19]. The time scale was then transferred to other phage genes through whatever segments of congruence were available. In this mode of thinking, the key elements are ancestors, which are definable as having existed at some point in time and having sent forward some population of descendants. In systematics, the analog of the ancestor is a higher-ranking taxon. In the current viral taxonomy, the higher-ranking taxonomy is based on the Baltimore classification, which was not constructed to reflect common ancestry. This creates a blind spot concerning how old the ancestors are to the phage clusters that do have common ancestry, and what is the history relating those ancestors to each other. With timetrees extrapolated into that blind spot, we seek to provide an objective matrix upon which to build models about the descent of phage gene modules and genomes.

A comparison of our previous analysis of *Pseudomonas* phage ΦRIO-1 [19] with the subsequent ICTV treatment illustrates the difference in perspective. Many of the ΦRIO-1 related phages have been grouped by ICTV into the family *Zobellviridae* based on the common content of numerous genes. The common ancestor of *Zobellviridae* tracks to ~1.5 Gya. The new ICTV families, where they have been created, generally correspond to ancestors in the 1.0–1.5 Gya range, so we think of that as an upper horizon for the ICTV system for correlating with common ancestry. For ΦRIO-1, deeper relationships were explored, working with only the easiest proteins for making deep trees (large terminase, portal, and tubeB). Those trees coalesced the ΦRIO-1 phages with other extended podoviral families of T7-like (*Autographiviridae*), P22-like (*Lederbergviruses*), and N4-like phages (*Schitoviridae*) in the range of 2.7–3.2 Gya. Above 1.5 and below 3.2 Gya, the similarity is difficult to detect, and information is therefore very sparse. The ΦRIO-1 study found that ΦRIO-1 had points of distant similarity to T7, and less so to P22, while N4 is dissimilar in the qualitative sense of not having a tubeB protein at all.

In this study, the most obvious distant similarity of ΦGT1 to a well-characterized prototype is to P22. We will use even more aggressive similarity detection to explore how consistently ΦGT1 components are related to an ancient P22-like ancestor, and if not, how the descent of ΦGT1 genes is mixed from different ancient ancestors. Working in this upper time range requires a different computational methodology than the typical ClustalW to tree program pipeline. The ΦRIO-1 study used recursive application of HMM methods to add ever more divergent clades to alignment, and used posterior scoring by HHpred in HMM-to-HMM comparisons to judge whether deeply diverged clades could be considered validly aligned, and, if so, in which segments. This practice is updated in this work at the point where alignments produced by other methods fail validation by HHpred HMM-to-HMM comparison. In that time zone, alignments of divergent clades were fused by enforcing the HHpred HMM-to-HMM alignment, thus clustering genes even more deeply into evolutionary history. ICTV is also incorporating HMM-to-HMM comparisons [20]. We differ from their approach by making a targeted effort to make robust broad-based alignments and HMMs for the proteins specific to the podo- (or myo- or sipho-) viral morphology groups, rigorously excluding recombination junctions from the sequences presented to the treemaking process, and evaluating the depth of common ancestors using the timetree paradigm.

With the abandonment of the morphological-tailed phage families, there are now no official names with which to describe clusters or hypothetical clusters of phages arising in the first 2 billion years of life. It is clear from the analysis of Hardies et al. [19] of the functional and sequence similarity of T7 and P22 tubeB protein that the podoviral ejectosomal mechanism was in existence by ~3.2 Gya. In a taxonomy designed to describe the full history of tailed phages, taxa dating to this period where bacteria form a small number of phyla would be phage phyla, and ejectosomal podoviruses would be one such phylum. From this molecular phylogeny perspective, “ejectosomal” podoviruses means all viruses that descended and retained the requisite gene set from the ancestor that invented the ejectosomal mechanism in the first place. We specify ejectosomal podoviruses rather than just podoviruses as the relevant universe of viruses to consider for this study to exclude phi29 and all related nonejectosomal podoviruses. These have different ancestry and functionality of their tail genes. Prospective subdivisions of the ejectosomal podoviruses in the 1.5 to 3.2 Gya range would be provisional podoviral classes or subclasses. These definitions can merge relatively gracefully with the ICTV system that becomes relevant below 1.5 Gya. It is helpful to have terms to mark these two distinctive epochs of time, so we will call 0–1.5 Gya the Blastoscene, and 1.5–3.8 Gya the Tailoscene. To avoid proposing and arguing about yet another naming system, we will name our ancestors of interest by the time-honored practice of naming one or more well-known descendant prototypical phage, but clarify by adding the estimated time of the common ancestor.

Another distinction of the timetree approach is that it allows a comparison of the timing of phage gene lineage splits with the timing of taxon differentiation in the host phylogeny. There is much discussion in the literature about phage mosaicism. Phages swap genes with other phages. There is a tendency to apply the term “horizontal transfer” to every gene newly acquired by a phage. For example, Mavrich and Hatfull [17] follow this convention. To us, a transfer from a source in a far away host taxon has different selective and mechanistic implications than picking up a gene from a phage descending in the same host range. With a time scale, we can distinguish those processes. We will use the term “reassortment” to describe the common shuttling about of genes among phages in the same host range. We will reserve the term “horizontal transfer” for a situation where the phage gene had to at some point move horizontally with respect to the host phylogeny.

This paper will focus on the descent of the ΦGT1 core structure genes. One peculiarity of the ΦGT1 genome is that HMM searching reveals two adjacent divergent homologs of the gene for the protein called tubular tail A (tubeA) in T7 and head completion protein, gene 4, in P22. This is the protein in the tail to which the tail fibers attach. We find that an ancestor with the ΦGT1-like dual tubeA structure gave rise to a large temperate phage family in *Rhizobiales*. The expansion of the dual tubeA family was tracked in *Rhizobiales*, along with several horizontal transferants cast off by it, including the path to ΦGT1. The origin of this dual tubeA structure was attributed to a recombination that occurred ~0.7 Gya between two more ancient lineages that both had the typical P22 structure module gene order. One ancestor descended from a LUZ24/phiEco32/alphaproteobacterial common ancestor ~1.56 Gya and donated the head structure genes and one copy of tubeA. The other ancestor descended from a Mx8/*Brucella* phage common ancestor ~1.25 Gya and donated the other copy of tubeA and the remaining tail module. The structural gene modules of both the LUZ24 and Mx8 ancestors have common ancestry with P22 >> 2.0 Gya.

## 2. Materials and Methods

### 2.1. Related Prophages

The search for related intact prophages started by searching completely sequenced bacterial chromosomes for close homologs to the ΦGT1 tubeB protein. TubeB was keyed upon to focus on podoviruses. To find an uninserted version of the same chromosome, the DnaE gene in that chromosome was used to key a BlastP search to find a collection of sequenced strains that were very closely related to the one containing the prospective prophage. A region of +/− 100 kb from the query chromosome was used to key a BlastN search of the DNA from those closely related chromosomes reported in the “-outmt 4” aligned format, and the gene annotations from the query sequence were transferred to the alignment. An ideal result would be a gap of ~40 kb starting in one or more closely related genomes within a tRNA gene adjacent to a gene labeled as an integrase in the query chromosome such that the tRNA gene was partially duplicated during the phage insertion. This pattern makes use of the frequent occurrence of phage integrases that use a host tRNA gene as the host attachment site and a partial version of the tRNA gene as the phage attachment site [21]. Prospective intact prophages were then examined for a full complement of core genes and a lack of genes disrupted by nonsense codons or frameshifts.

### 2.2. Homolog Candidates and Alignment with the UCSC Sequence Alignment and Modeling System

Alignments were made and extended to highly divergent sequences similar to the method described [19]. Typically, sets of prospective homologs were found by Psi-Blast searches starting from the ΦGT1 gene or from candidates for homologs in distant phages suggested by similarity in gene size and genomic position, or other criteria. Psi-Blast [22] was installed locally from the NCBI Toolkit so that databases other than the standard NCBI nr database could be easily searched, for example, nr fused to the metagenomic env_nr, or databases reduced to just tailed phages, or just podoviruses, or just the proteomes of selected individual phages. Psi-Blast iterations were run until either convergence or the accumulation of obvious nonhomologous sequences. The proposed homologs will be screened for significant similarity by a sensitive HMM-based method in the next step, allowing this initial step to be optimized for high sensitivity with less concern about the inclusion of false positives. Homolog candidates were then screened and aligned by the UCSC Sequence Alignment and Modeling system (SAM ver. 3.5) [23,24] obtained from Kevin Karplus and Richard Hughey [https://users.soe.ucsc.edu/~karplus/projects-compbio-html/sam2src/ (accessed on 16 December 2022)]. Although we have also used more common alignment systems, SAM has proven particularly useful in its property of iteratively screening a prospective homolog set to include only sequences with statistical similarity to the developing HMM, and then incorporating those sequences and reoptimizing the HMM and its underlying alignment for the next iteration.

### 2.3. Alignment Validation

After preliminary tree construction using neighbor-joining in PAUP [25], or MrBayes, sequences forming the most divergent clades were realigned independently and compared to each other by HHpred [HHsuite ver. 2.0.15 installed locally; most recent version obtainable at https://github.com/soedinglab/hh-suite (accessed on 27 March 2022)] [26]. Models were built including secondary structure prediction with the included addss utility. Models were calibrated against the scop70_1.72 HHpred model library and searched against each other one to one by hhsearch with one designated as the query and the other designated as the database. This method produces posterior residue alignment confidence scores. Strings of confidence scores of mainly 7, 8, or 9 were taken to indicate regions of confident alignment. The HHpred alignment was compared to the SAM alignment to confirm that the two methods were in agreement in the confidently aligned segments. If there was no confidently aligned segment, the divergent clade was removed from the analysis. If removal of the non-confidently aligned segments did not alter the tree, they were left in place, since they presumably improve confidence in the lower parts of the tree. If HHpred found a confident alignment and SAM failed to agree, subfamily alignments were fused by forcing a merged alignment to adhere to the HHpred result. Whenever this happened, it is indicated in the legend to the supplementary tree figure. In principle, the program Clustal Omega conducts this same operation. However, in practice, we found processing alignments by SAM to be more successful in sweeping gaps out of regions with predicted secondary structures.

### 2.4. Availability of Alignments and HMMs

Alignments and HMMs from this study are available at https://Stephen-Hardies.github.io (accessed on 10 May 2023). Some of these protein families may eventually appear in Pfam, but in many cases, the criteria used by Pfam contradict the criterion we used for forming the alignment. Pfam prioritizes confident family assignment in a large database search and seeks to limit confusion caused by related families by making separate models for each. We prioritize the HMM as a tool to align distant homologs, which requires all the related subfamilies to be present in the same alignment. If used blindly, say to annotate an entire bacterial genome, these broader models are more prone to raise false positives. However, when used for alignment of genes and treemaking with validation procedures, false positives tend to be much less of a problem. The HMMs provide a direct method to align any collection of related sequences using either hmmeralign or Clustal Omega.

### 2.5. Treemaking

For the final tree analysis, a small subset of sequences was extracted from the larger alignments made above. Trees were constructed by MrBayes (ver. 3.2.6) [27] with the BLOSUM replacement matrix as the amino acid prior, a four-category gamma correction, and the independent gamma rates relaxed clock model, also called the “white noise” model [28], with 4 chains and 1,000,000 generations and 50% reserved for burnin, unless the convergence statistics indicated a need for more generations to achieve convergence. Consensus trees were compiled by the sumt tree utility with the “allcompat” option, and displayed and scaled using Figtree [http://tree.bio.ed.ac.uk/software/figtree/ (accessed on 8 December 2015)]. Divergence times for host taxa for comparison were from the TimeTree website [http://timetree.org (accessed on 3 May 2023)] [29]. For comparison to maximum likelihood, the same alignment was analyzed by MegaX [30].

### 2.6. Tree Quality

Several controls were employed to guard against low-quality trees. If subtrees produced from separate domains or of the sequence arbitrarily divided in half did not appear mutually congruent, the tree was rejected, and a search was conducted for the underlying recombinant sequence(s) and recombination points. The tree was then remade without the inclusion of recombination junctions. If addition or subtraction of sequences made the tree incongruent with its former self, the tree was rejected and a search was conducted for the underlying cause, which might be intragenic recombination, substantial poorly aligned segments, or branches or clades evolving at a systematically different rate. The tree was then remade without the problematic segments or sequences.

### 2.7. Tree Scaling and Congruency

The large terminase tree was made with a selection of far-diverged terminases and the root set at 3.8 Gya—the approximate origin of life on Earth (3.7–4.2) [31]. For two protein trees to be considered candidates for congruence it was expected that their trees could be aligned with consistent topology so that there were a series of nodes for which the median node height of each fell within ~1.4 times the 95% Height Posterior Density (HPD) of the other tree. The 1.4 multiplier was an effort to account for the joint uncertainty of both trees. Collections of prospectively congruent nodes in the phiEco32/LUZ24 family and ΦGT1 family derived as explained in Section 3.3 are given in Table S1A,B. Averages node heights are given excluding any node that appears to fail the congruence test with the others or for which the quality of information is otherwise less than the others. The time scale of each timetree was fine-tuned to maximize agreement with these averages. Candidate trees were first scaled to make the highest of the candidate nodes of equal time to the value from Table S1A,B. Nodes that failed the congruency test with the reference values were excluded. The deviations of each remaining candidate congruent node from the average of the collection were normalized by 1/4 the 95% HPD, essentially expressing them in numbers of standard deviations. The deviations were then summed, and the time-scale factor was adjusted to make the sum equal to zero. Nodes excluded or additional calibration points added are listed in legends to the supplementary tree figures.

### 2.8. Mass Spectrometry

Mass spectrometry was carried out at the National Instrumentation Center for Environmental Management, Seoul National University. RP-nano LC-ESI-MS/MS analysis was conducted using a Thermo Scientific Q Exactive Hybrid Quadrupole-Orbitrap instrument (Thermo Scientific, CA, USA) equipped with Dionex U 3000 RSLCnano HPLC system. Fractions were reconstituted in solvent A (Water/Acetonitrile, 98:2 v/v, 0.1% Formic acid) and then injected into the LC-nano ESI-MS/MS system. Samples were first trapped on an Acclaim PepMap 100 trap column (100 μm × 2 cm, nanoViper C18, 5 μm, 100 Å, Thermo Scientific, part number 164564) and washed for 6 min with 98% solvent A at a flow rate of 4 μL min^−1^, and then separated on an Acclaim PepMap 100 capillary column (75 μm × 15 cm, nanoViper C18, 3 μm, 100 Å, Thermo Scientific, part number 164568) at a flow rate of 300 nl min^−1^. The LC gradient was run at 2% to 35% solvent B (80% acetonitrile, 0.1% formic acid) over 30 min, then from 35% to 90% over 10 min, followed by 90% solvent B for 5 min, and finally 5% solvent B for 15 min. The Orbitrap analyzer scanned precursor ions with a mass range of 350–1800 m/z with 70,000 resolution at m/z 200. Xcaliber software (version 3.1) was used to collect MS data and Mascot (Matrix Science, London, UK) was used to search the spectra against a database of predicted ΦGT1 proteins concatenated with the Swiss-Prot database. Mascot results were correlated with X! Tandem with Scaffold (Proteome Software, Inc., OR, USA).

### 2.9. Ascertainment of Membership in Morphological Families

In support of statements that a lineage is either podovirus-specific or contains a mixture of podo-, sipho-, and myoviruses, a problem has arisen in that NCBI has begun expunging the morphological family names from phage entries. Hence one can no longer research the morphotypes of a collection of protein accessions mapped to a tree by any operation performed on the current NCBI database. As a workaround, we restrict a preliminary survey to phages present in a saved back copy of the NCBI database as of 31 May 2019, before the morphotype information was expunged. The finding was only considered definitive if an associated paper cited the EM examination. The annotation of morphological family types for metagenomic phage sequences was ignored.

### 2.10. Neighborhood Analysis

To determine if a gene is consistently located in the same gene neighborhood, we performed the following analysis: NCBI was interrogated to find the nucleotide sequence encoding each gene in the tabulated family of the query sequence. Protein sequences defined as features within the nucleotide sequences were retrieved corresponding to a neighborhood defined as some number of genes upstream and downstream. The protein sequences were formed into a library with each entry carrying its original annotation plus its position in the number of genes upstream or downstream relative to the query sequence. The annotation of the sequences in this library was updated with HMM search results for landmark genes in the neighborhood of the original query. The frequency at which homologs of the landmark genes fell within the neighborhood of homologs of the original query could then be directly examined.

### 2.11. Clade Trees

The trees in Section 3.8 were designed to reveal how the abundance of the temperate phage families fluctuated in different host taxa and taxa inferred from metagenomic data. For prophages, the analysis was confined to completely sequenced and assembled bacterial chromosomes that were retrieved from NCBI 5/2019 and curated to incorporate a full taxonomic description within each protein definition line and to avoid plasmids. It had 17,457 genomes. This was intended to facilitate counting the number of sequenced chromosomes that yielded the number of prophage homologs found in any taxonomic envelope. The metagenomic collection was retrieved from NCBI 10/2020 with an Entrez query including “uncultured phage” in the definition line. It had 3187 genomes. Search and alignment by Psi-Blast and SAM were conducted as in Section 2.2 with the inclusion of a blast-formatted version of this metagenomic collection. Clades were defined by preliminary neighbor-joining analysis. Exemplars from each clade were subjected to MrBayes analysis to create the time scale for the clade trees. The final tree construction was as described in the figure legend.

### 2.12. Replacement/Synonymous Analysis

Protein sequence alignments were converted to nucleotide alignments using pal2nal [32]. Replacement and synonymous changes were counted and normalized by the number of sites using the DNAStatistics module of the BioPerl Align package.

## 3. Results

### 3.1. The ΦGT1-Related Prophage Collection

The analysis was aided by including prospective prophage sequences related to ΦGT1. For some purposes, we thought it beneficial to focus on intact prophages. This was carried out by looking for ΦGT1-related genes present in a clean approximately 40 kb insert relative to closely related chromosomes and ideally surrounded by identifiable attachment sites as described in Section 2.1. Clusters of prophages were targeted in the heavily populated *Sinorhizobium* host taxon, and then additional prophages were targeted in host taxa including far-off locations that would require a horizontal transfer to reach. Table S2A lists a series of prospective prophages used in the study. The two *Sinorhizobium* lineages marked “m” and “f” are the closest match to the degree of divergence analyzed by Mavrich and Hatfull [17] and described further below. These can be thought of as *S. meliloti* and *S. fredii* lineages although they are not completely isolated, which is taken up further in Section 4.4. The *Sinorhizobium* “f” cluster was problematic in that we could not identify an integrase or an attachment site. However, a replacement/synonymous analysis on the terminase, portal, and tubeB genes of the “f” cluster revealed similar selective pressure as for the “m” cluster (Table S2B). Hence the “f” lineage has some of the properties of a lineage of actively propagating phages, although a full understanding of its character is still elusive.

### 3.2. Summary of the Ancestry of the ΦGT1-like Core Structure and Morphogenesis Module

**Figure 1:**
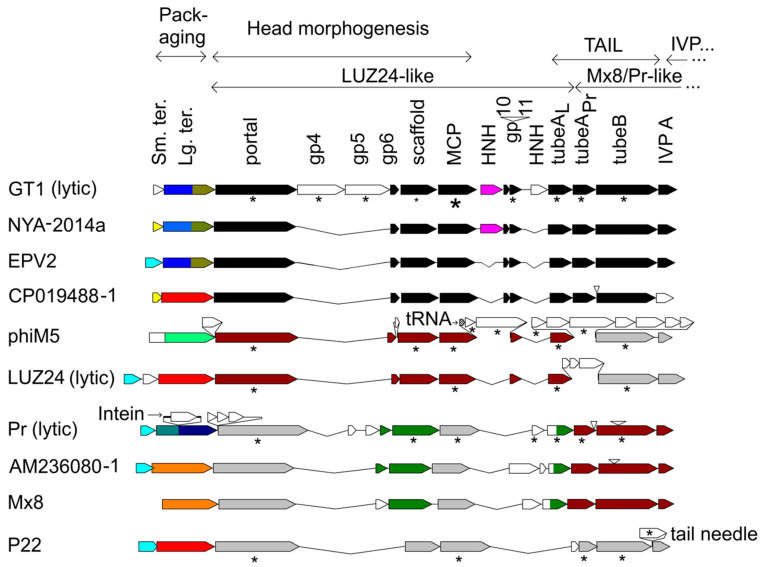
Comparative gene arrangement of core structure modules in ΦGT1 and selected related podoviruses. The phages are temperate unless indicated. The two tubeA paralogs are labeled tubeA_L_ and tubeA_Pr_ to indicate the one from the LUZ24 lineage and the one from the Mx8/Pr lineage. Genes indicated in black, brown, or gray are estimated to have common ancestry as follows: black ≤ 0.7 Gya; brown 1.0–1.5 Gya; gray > 2.0 Gyr (see text). Other colors indicate similarity clustering not congruent with the patterns shown in black, brown, and gray. Proteins found to be present in the virion are indicated by an asterisk (*). For ΦGT1, the asterisks are scaled to indicate three different abundance groups as determined by spectrum counts (Table S3). Sources for presence in virion are LUZ24 [9]; ΦM5 [8]; Pr [7]; P22 [33]. The gene labeled IVPA is homologous to P22 gene14, and more distantly to T7 IVPA.

Starting with Psi-Blast and HHpred searches, proceeding through HMM family expansion, and ending with detailed timetree analysis, two congruent sets of genes were identified: one matching first to ΦM5 and then LUZ24-like phages, and one matching first to *Brucella* phages, and then Mx8 and 45A6. From the timetrees describing these two sets of genes, a model was developed for the descent of the genes of the ΦGT1 structure and morphogenesis module starting from the most ancient known podoviral common ancestors. Figure 1 gives a comparative map of selected phages and prophages in the region from the terminase genes through the core head and tail genes. It shows the segments derived from the LUZ24-like lineage and from the Mx8/Pr-like lineage, and summarizes mass spectrometry results where available.

Figure 1 focuses on the most coherently descending genes of these genomes. Excluded are the nonstructural modules and the tail fiber genes and most of the internal virion protein genes. Internal virion protein genes and then tail fiber genes are generally located to the right (except some are interspersed with tubeA and B in ΦM5) and difficult to place on a tree due to higher divergence and/or higher mosaicism. In Figure 1, the patterns shown in brown illustrate how the recombinant dualA arrangement corresponds to the two anciently P22-like lineages that gave rise to it. As will be illustrated in detail in the trees below, the unrecombined representatives of those lineages include ΦM5, a member of the LUZ24-like lineage with common ancestry just before the recombination, and Pr and AM236080-1 which are members of the Mx8-like lineage splitting off just before the recombination.

The large and small terminase subunit genes exhibit considerably more mosaicism relative to the core structural genes than the structural genes do among themselves. The tentatively assigned small terminase of ΦGT1 has no detected sequence similarity to any characterized small terminase. The basis of the assignment is that wherever homologs are found scattered through prospective prophages, they are always upstream of a large terminase gene (Section 2.10), and we have yet to see a case where a homolog of this gene is present and there is a better candidate for small terminase. We suspect that the small terminase is a focus of diversity selection which drives enhanced recombination of the large terminase by linkage disequilibrium. The idea is by analogy to tail fiber mosaicism, wherein the selective pressure is thought to mainly be for switching the antireceptor at the C-terminal domain of the fiber as part of a coadaptive evolutionary struggle with the host. The C-terminal domain is frequently replaced from one phage to the next. The N-terminal domain of the tail fiber is seldom replaced in a phage group, presumably because its property of binding to the tail hub is conserved. Recombination points that replace the C-terminal domain fall at a variety of positions along the fiber, so intermediate parts of the fiber are replaced with intermediate frequency, basically forming a diversity gradient. In the case of the terminase module, we postulate that the small terminase functions to identify and bind the phage concatemer for packaging as is the case for lambda small terminase [34]. In temperate phages, there would be a selection for diversifying phage lineages occupying the same hosts to select their own DNA for packaging. The recombination points that switch out the small terminase gene wouldn’t necessarily always fall at the boundary of the small terminase gene. Sometimes, in the process of exchanging the small terminase, the large terminase would be exchanged also. This might provide a rationale for why large terminase has higher mosaicism than the adjacent head structure genes without having to postulate a direct benefit for exchanging it (see Figure 1).

Mass spectrometry results are summarized in Figure 1 and tabulated in Table S3.

### 3.3. Construction, Calibration, and Quality Control of the Timetrees

Timetrees of large terminase and each of the conserved proteins from Figure 1 were made as described in the method section. Our procedure is inspired by the bacterial phylogenetic trees of Battistuzzi et al. [31]. They start by finding individual core proteins that appear to be mutually congruent, then concatenate them and make a single tree for presentation. Given that the number of topological disruptions of congruency among phage genes is relatively high, we do not concatenate the sequence to make one tree. Instead, each gene is evaluated independently and sequences of nodes that are mutually congruent are identified for use in producing a mutually consistent time scale. Selected trees are shown below and these and trees for each of the other structural proteins appear in Figures S3–S12 with additional documentation. Among all these trees, the topologies of the LUZ24 module clades are similar to each other, and the topologies of the Mx8/Pr clades are similar to each other.

When working these trees, it became clear that recombination wasn’t only a problem at sites between genes. The large terminase shows several intragene recombinants where the N-terminal domain alone makes a much different tree than the C-terminal domain, and at least one case (in Pr-related phages) where there is a recombination point within the N-terminal domain (Section 3.4). Including these recombinant sequences in the tree build in some cases confused the topology, but in all cases shifted node heights outside of the calculated uncertainty intervals. Similarly, it was found that including bacterial chromosomal homologs of the terminase without paying attention to removing pseudogenes would sometimes cause nodes to shift beyond the calculated uncertainty intervals. Several controls designed to detect and eliminate problems of this kind are given in the methods. After removing those issues, some controls were carried out on the quality of the timetree time scale. These are described in detail in Figures S1 and S2. In short, our single protein timetrees have a similar capability to extrapolate a time scale as exhibited by the bacterial phylogenomic results reported [31]. Additionally, there is a saturation effect for the more rapidly diverging proteins such that distance to the most ancient nodes tends to be underestimated. Fortunately, we found the large terminase tree, which is the source of our time scale, to have a minimal problem with this effect as judged by successfully timing the T7 RNA polymerase link to mitochondrial RNA polymerase [35] to the mitochondrial endosymbiosis at ~1.9 Gya [36]. Additionally, we find that the Bayesian node age estimates for the most recent nodes are systematically exaggerated, requiring maximum likelihood estimates to give correct registration with time in that zone.

### 3.4. Large Terminase and Portal Trees

**Figure 2:**
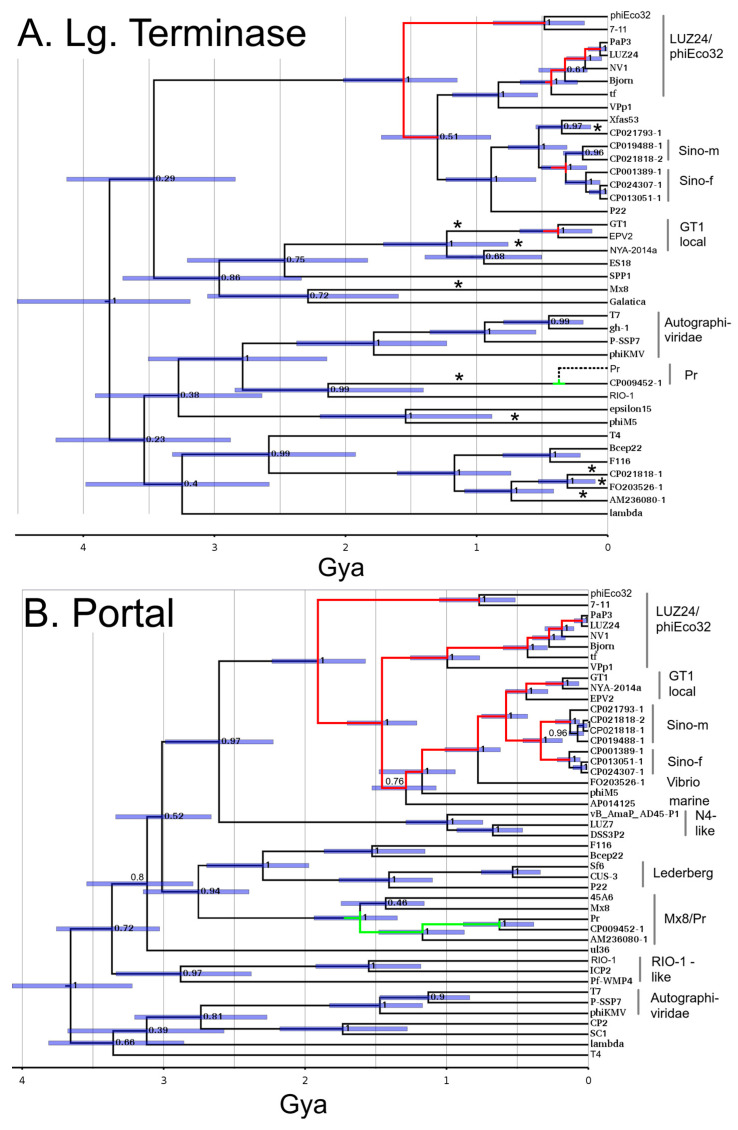
Timetrees for (**A**) large terminase subunit, and (**B**) portal protein with relatives of ΦGT1 and selected other phages to fill out the tailed phage tree space. On the portal tree, nodes that are generally congruent across the LUZ24 module are bisected by a red line, and across the Mx8/Pr module by a green line. On the large terminase tree, only the portion of these nodes that are not disrupted by the replacement of the large terminase relative to the structural genes are color coded. Numbers at the nodes indicate posterior topological support, and the node bars indicate the 95% node Height Posterior Density (HPD) of each node. The Pr lineage below the node with CP009452-1 is shown as a dashed line for large terminase because the large terminase in Pr and the other *Brucella* phages is an intragenetic recombinant, thus requiring a separate tree to avoid the recombination junction. Asterisks mark branches that have experienced recombination relative to the structural module genes. Additional details and commentary can be found in the tree supplement.

Figure 2 shows the large terminase timetree calibrated to set the root at 3.8 Gya, and the portal timetree calibrated to maximize congruency with the large terminase tree. As can be anticipated from Figure 1, the large terminase has recombined several times relative to the structure gene modules. These recombinations disrupt congruency with the structural genes, thus complicating the transfer of the time scale. Fortunately, the LUZ24/phiEco32 cluster of lytic phages did not have this problem. Preliminary trees in this clade showed congruency across all the structural genes including the large terminase with few exceptions. The degree of consistency of a selected set of test nodes relative to phiEco32/LUZ24 set to the height from the terminase tree is given in Table S1A.

The ΦGT1 family nodes appear on the same trees as the LUZ24/phiEco32 family nodes. Although the various ΦGT1 genes cannot be usefully scaled against the ΦGT1 terminase lineage, they do appear reasonably congruent in the context of their LUZ24/phiEco32 ancestors and were scaled accordingly. The degree of consistency of a selected set of ΦGT1 family test nodes scaled in this way is given in Table S1B. The average node heights across the structural genes tabulated in Table S1A,B provide an abundant array of time markers against which time calibration of each final timetrees can be fine-tuned as described in Section 2.7.

The portal tree was calibrated by points of congruence with the large terminase tree, mainly within the LUZ24 clade. Our criterion for congruence is for a node to fall within 1.4 times the 95% HPD of the same node on another tree as explained in Section 2.7. The portal is the first of 7 genes found to have mutual congruency which includes the first tubeA gene (below), scaffold, major capsid protein, and three other small genes of unknown function found in the supplementary figures.

The Mx8/Pr clade makes its first appearance in the portal tree as a cluster of the *Brucella* phages (represented by Pr), *Ruegeria* phage 45A6, Mx8, and AM203080-1, a prospective prophage in a large family in *Rhizobium leguminosarum* selected specifically to flesh out this family. Mx8 is a temperate phage of the deltaproteobacterium *Myxococcus xanthus*. It is temperate and has been used to mediate general transduction in *Myxococcus* [13]. It is the only member of the ICTV taxon *Myxoctovirus*. The Mx8/Pr clade is not apparent in the large terminase tree because most of its representatives had recombined to incorporate a large terminase from a different large terminase clade. The only node judged to be congruent between the large terminase tree and the various structural protein trees within the Mx8/Pr clade is that of Pr with prospective prophage CP009452-1.

To establish some context relative to the heavily characterized P22-like viruses, three members, all currently clustered at the genus level (*Lederbergvirus*), are mapped covering the diversity of the genus as exposited [37]. The age of the common ancestor of the portal variants carried in P22-like phages is much, much older than the common ancestor of the host genera from which they are derived. On the other hand, the age of the common ancestor of the tubeB variants of the P22-like phages (Section 3.6 below), is roughly consistent with having developed in the hosts with which the phages are associated. That is a curious observation in that it suggests that something akin to a selective sweep affected tubeB in *Lederbergviruses*, although no such trend appears in the other podoviral clades examined here.

### 3.5. The Dual TubeA Tree

**Figure 3:**
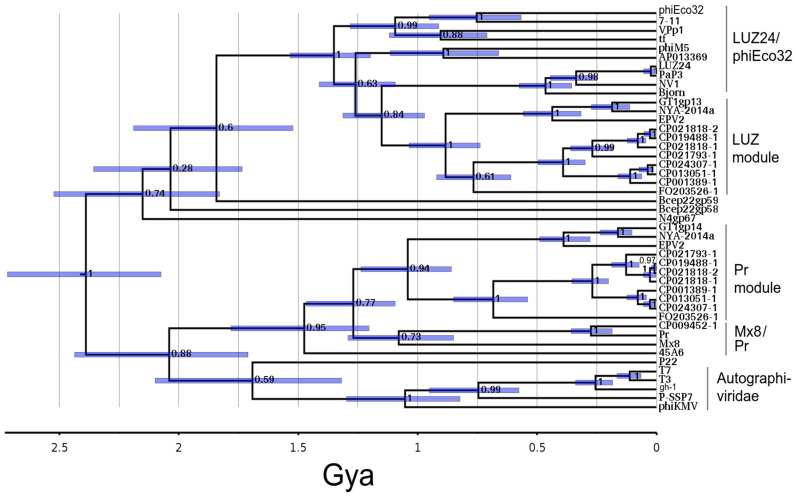
Timetree of the LUZ24-like tubeA and Mx8/Pr-like tubeA paralogs and selected homologs.

Figure 3 shows both the LUZ24-like and Mx8/Pr-like tubeA proteins on the same timetree. The Mx8/Pr-like tubeA is the first of three genes showing a congruent pattern of descent, including tubeB (below) and the IVPA homolog (Figure S11). ΦGT1 tubeA_L_ and tubeA_Pr_ are extremely divergent by any criterion. In the initial Hhpred survey, tubeA_L_ matched so weakly to the established tubeA models that we did not take it seriously as a second tubeA paralog until we realized that its homologs were the only tubeA candidates in the entire LUZ24-like collection of phages. The position of the LUZ24-like tubeA clade as further out than the ancestor relating T7 to P22 appears to be a valid result. That result conflicts with the expectation created by tubeB that P22 and T7 are the most divergent of the ejectosomal podoviruses and complicates the picture of how the initial radiation of the podoviruses occurred. However, the ΦGT1-like dual tubeA arrangement did not arise from a recent duplication and specialization of a tubeA gene. Rather, it was created by a fusion between genomes with the most divergent possible tubeA paralogs. In the process of exploring this, it was noticed that Bcep22-like phages also have two adjacently encoded tubeA paralogs, and those were added to this tree. The Bcep22 dual tubeA was also created by a fusion drawn from among the most ancient possible paralogs and was completely independent of the ΦGT1-like fusion.

### 3.6. TubeB Tree

**Figure 4:**
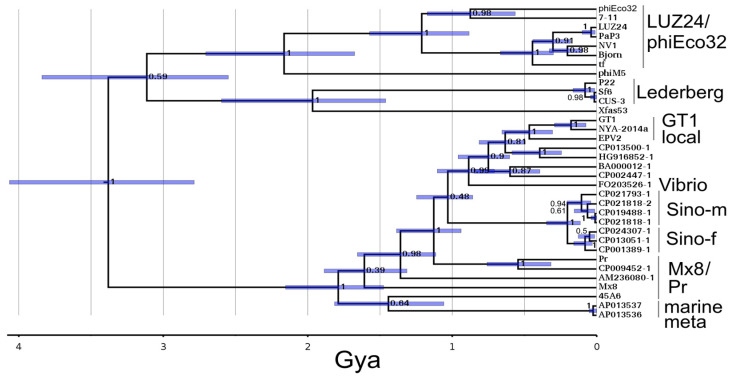
Timetree of ΦGT1 tubeB with selected homologs showing clustering with the Mx8/Pr clade.

The tubeB timetree is shown in Figure 4. Hardies et al. [19] noted that T7 tubeB and P22 tubeB (aka. Head completion protein gp10) were only reliably aligned in a central segment and had length differences in the N- and C-terminal portions. All the sequences shown in Figure 4 have the length characteristics of P22 tubeB. However, alignment checking with Hhpred indicated that there was still only reliable alignment across the central domain, so only that segment was used to make this timetree. The conserved portion of the sequence corresponds to four of the six domains of the “beta-propeller” section of the structure [38]. The conserved section lines the interior surface of the tail tube and has no exposure to the external surface of the tail structure. Hence the lesser conserved segments do not necessarily indicate intragene domain recombination.

If the T7 clade is added, it joins unresolved with the midpoint root. Therefore, if length variation is given weight, then these are all P22-like just underneath the junction with T7-like phages as an outgroup. If only sequence divergence is given weight, T7-like, P22-like, Mx8-like, and LUZ24-like are four separate clades of ancient origin. There are a few additional prospective prophages shown linked to the ΦGT1 local clade of tubeB that were not included in all the trees. These are discussed in Section 3.8 below.

### 3.7. Comparison to Mavrich and Hatfull Treatment

The most thorough treatment of gene reassortment in phages was published by Mavrich and Hatfull [17]. They were able to distinguish two classes of phages one of which was ~10-fold more recombinogenic than the other, with the two classes mainly corresponding to temperate and lytic phages, respectively. They measured the degree of recombination as the fraction of genes that cannot be aligned at the nucleotide level at a given average nucleotide identity (ANI) of those genes that can still be aligned at the nucleotide level. Their results could be summarized as the high recombination class having replaced 70–95% of their genes when the residual genes were reduced to 70% ANI, whereas the low recombination class replaces 40–60% of their genes by that time. The only well-populated node in our study young enough to compare at the nucleotide level is the node connecting the *Sinorhizobium* “m” and “f” lineages. They join at about 70% ANI with 83% replacement which is squarely in the Mavrich and Hatfull high recombination zone. In our trees, the Bayesian estimate of the node height corresponding to this division ranges from 0.2 to 0.4 Gya. This is in the time zone in which we believe the Bayesian estimates to be exaggerated (Figure S1 and associated text). The tubeB tree was run back through Mega to arrive at a maximum likelihood estimate, which was ~77 Mya, which we believe to be more accurate. The genes still aligning at the nucleotide level between “m” and “f” lineages correspond exactly to the core structure genes analyzed in this work. Essentially none of the genes of the nonstructural module match by BlastN above ~77 Mya. Using Blastp to extend sensitivity does detect matches among our test set of prospective prophages, but each gene in its own idiosyncratic selection of prophages. Comparison of the prophages within the “m” and “f” clades more closely related than 77 Mya revealed that although the Sino “f” lineage seemed relatively resistant to recombinational exchanges, the Sino “m” lineage was affected by the replacement of large blocks of genes, similar to the pattern initially described [39]. The Mavrich and Hatfull analysis is therefore dominated by the recombinational churning of nonstructural genes, while the core structural genes simply diverge past the point where they can be aligned at the nucleotide level, but appear to have diverged relatively coherently from much more ancient times by protein sequence comparison. Essentially any of our clade comparisons, if matched with BlastP or Psi-Blast show the same 15% of genes conserved in the structure module, and the rest of the gene organization is mostly scrambled beyond recognition by recombination. We suspect that the generalization of 10-fold higher reassortment of temperate over lytic phages also applies to the structure and morphogenesis module but as a ratio of substantially lower recombination rates in both classes of phages. This is most easily noticed in the number of recombinations affecting large terminase relative to the structural genes (Figure 1).

### 3.8. Host Distribution of the LUZ24-Like and Mx8/Pr-Like Structure Modules and Relationship to Metagenomic Oceanic Phage

**Figure 5:**
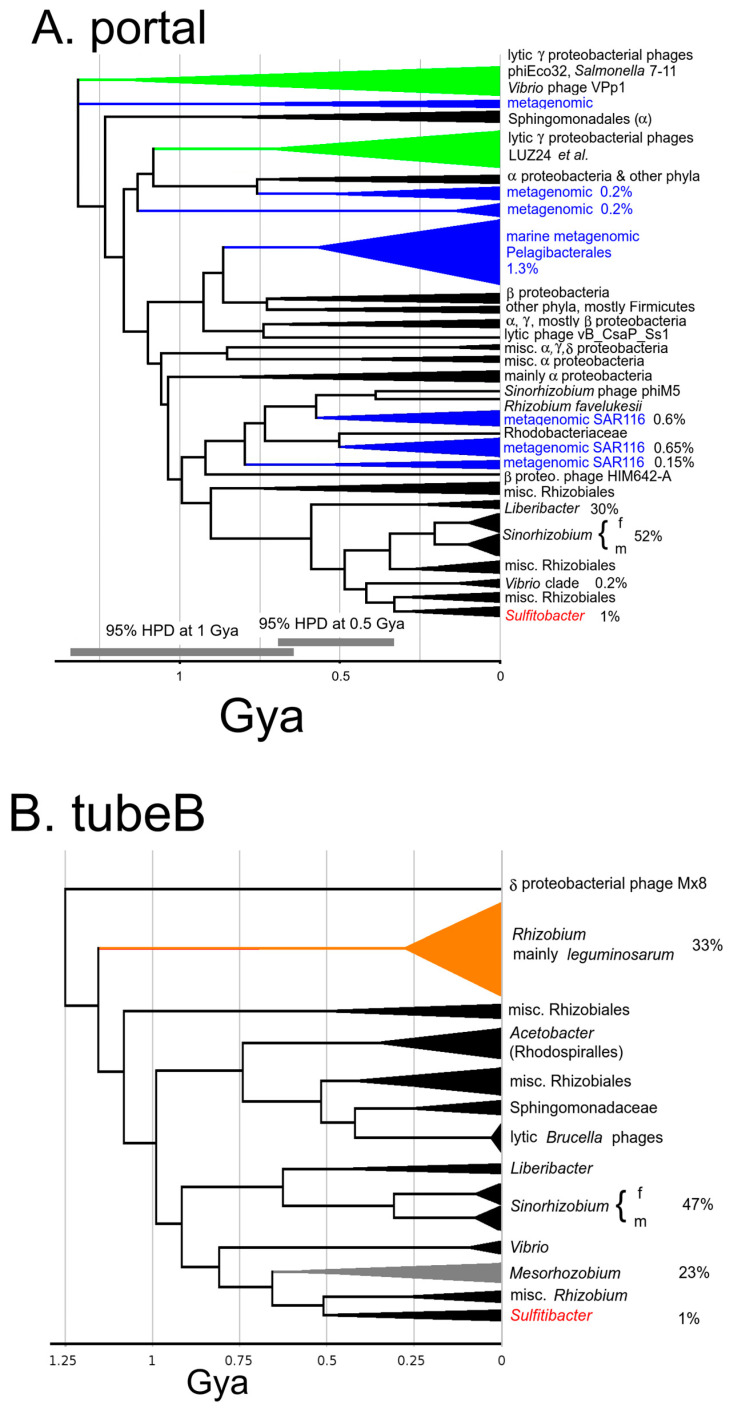
Depiction of clade sizes and host locations of (**A**) LUZ24-like modules as represented by the portal, and (**B**) Mx8/Pr-like modules represented by tubeB. All Psi-Blast matches out to ~1.4 Gya in sequenced bacterial chromosomes or metagenomic data are included as described further in Section 2.11. Clades defined by a neighbor-joining tree are depicted as a triangle and grafted onto a MrBayes tree made from exemplars from each clade. The triangles are roughly scaled corresponding to abundance and labeled by the taxonomy of the host bacterium. For taxa of interest, percentages are given to indicate the fraction of fully sequenced genomes of that taxon or the fraction of total metagenomic phage sequences that contain the indicated module. Blue indicates metagenomic clades; green indicates gammaproteobacterial lytic clades, orange indicates the most abundant population of Mx8/Pr representatives, which are prophages in *Rhizobium* particularly concentrated in *R. leguminosarum*; Red is the ΦGT1 local clade which consists of three prophages in *Sulfitobacter* and the EPV2 metagenomic construct. The percentage of *Sulfitobacter* genomes occupied is given. These trees are expected to be less accurate than the trees with small numbers of carefully curated sequences. Typical 95% HPD intervals for two different time ranges are marked on panel A.

Figure 5 explores how the descendants from the LUZ24 and Mx8/Pr ancestors at ~1.4 Gya have become distributed in the sequenced fraction of the bacterial world. One thing that should be immediately apparent is that the temperate phages distribute very unevenly, heavily populating certain taxa, such as *Sinorhizobium*, and lightly populating others, such as *Sulfitobacter*.

One feature of the LUZ24 lineage clade tree is that the LUZ24 lineage left a major contribution within oceanic metagenomic sequences. These metagenomic lineages split off before the fusion with the Mx8 lineage (Figure 5A, blue clades). A larger version of the analysis in Figure 5B extended to include the LUZ24-like tubeB genes similarly showed metagenomic clades arising at these times. The presence of the tubeB gene verifies that these LUZ24-related metagenomic sequences are podoviruses, even though they are almost all misannotated as siphoviruses. Following up by screening all sequenced metagenomic phage genomes with an HMM able to detect all tubeB genes found that 24% of them have a tubeB gene, and are therefore podoviruses. The major LUZ24-like metagenomic clades found in this work contained accessions of sequences analyzed by Mizuno et al. [3]. They organized their sequences into clades which they associated with bacterial hosts. The larger marine clade of Figure 5A was assigned to the largely unculturable alphaproteobacterial *Pelagibacterales* order and splits out at a time that would have been close to the inception of that order. Most of the other marine metagenomic finds in Figure 5A correspond to a lineage identified as infecting marine alphaproteobacterial clade SAR116 [3]. The SAR116 clade seems to have been the source of *Sinorhizobium* ΦM5 based on the topology of Figure 5A. Although ΦM5 is temperate and is grown in the same *Sinorhizobium* strains occupied by the ΦGT1-like prophages, there are no ΦM5-like prophages in the sample of sequenced genomes from *Sinorhizobium*. We only found one related prospective prophage in all *Rhizobiales*. Immediately after a horizontal transfer the transferred sequence is at an infinitesimally low frequency. It logically would take some time to sufficiently amplify so that it could be caught in a random sequence survey. Hence, a horizontal transfer from the heavily populated SAR116 clade could explain how ΦM5 could have become a temperate phage of *Sinorhizobium* and yet not be found in the sequenced genome survey. Unlike the LUZ24 module, the Mx8/Pr module made no major contribution to oceanic metagenomic lineages (Figure 5B).

Another feature of the clade tree in Figure 5B is that it fleshes out the passage of the dual tubeA lineage into *Rhodobacterales*. There are two clades in Figure 5B splitting off the tree during descent from the node with *Vibrio* (which includes FO235261-1 in Figure 4), one populating hosts in *Mesorhizobium* (which differs at the family level from *Sinorhizobium*), and one populating other *Rhizobium* species (which are in the same family as *Sinorhizobium*). In Figure 4, CP002447-1 and BA000012-1 represent the *Mesorhozobium* clade, and CP0013500-1 (*Rhizobium etli*) and HG916852-1 (*Rhizobium favelukesii*) represent the other *Rhizobium* clade. Further examination of these indicates that the other *Rhizobium* common ancestor with ΦGT1 has the dual tubeA architecture and represents the most direct donor from *Rhizobiales* family *Rhizobiaceae* to *Rhodobacterales* family *Roseobacteracea*. On the other hand, the *Mesorhizobium* clade does not have the dual tubeA architecture even though it is descended from the node representing the dual tubeA fusion. This would suggest that the dual tubeA genome remained in contact with descendants of its parental lineages and this case further recombined to remove the LUZ24 component and replace it with yet another clade from the Mx8/Pr lineage. This serves as a caution that our method of analysis focused on finding the path to our ΦGT1 target genome has probably ignored other recombinant trajectories occurring and leading off in different directions.

## 4. Discussion

### 4.1. Relationship of ΦGT1 to the Common Ancestral Podovirus

We have shifted our analysis of phage genomes to timetrees to have a more quantitative basis for examining congruence and to more clearly picture the early times during which the gene modules were established. Modules exhibiting coherent descent over the longest periods of time were prioritized for establishing an overall evolutionary framework of the family’s descent. The time scale was set to make the initial radiation of the packaging ATPase system at 3.8 Gya, consistent with the theory that phages were either involved in the invention of DNA and the transition from RNA to DNA world or appeared very soon thereafter [19]. There is a range of opinions on the timing of the origin of life (reviewed [31]). However, given that the uncertainty of the reconstructions covers about 1 Gyr at that depth in time, we have picked the 3.8 Gya number as a commonly quoted estimate that is approximately in the middle of the plausible range. The proposition is that this will allow us to place common ancestors in the phage tree into the context of bacterial time trees roughly within the uncertainty intervals shown by the node bars on the trees. The best support for this proposition is found in Figure S2, in which we used this system to time the mitochondrial endosymbiosis with a phage gene, and found an estimate consistent with the current consensus opinion of the time for that event.

On that time scale, the most anciently established trait of ΦGT1 is that it is a podovirus with an ejectosome. This kind of virus was established by 3.2 Gya and the form has descended maintaining a coherent collection of structural components. This kind of podovirus is most easily recognized at the sequence level by an encoded tubeB protein followed by candidate genes for the ejectosomal proteins themselves. By ~1.5 Gya the ancestors to ΦGT1 have coalesced into two families which each has become mainly committed to a specific structural gene module: the LUZ24/phiEco32 family and the Mx8/Pr family. These two families subsequently recombine to make the ΦGT1-like genome. Both families feature more P22-like than T7-like tubeB proteins by HMM scoring, but by tree analysis diverged in the early Tailoscene soon after or at the same time as the T7/P22 split. Among the other proteins traced into this time zone, the most consistent feature is that the LUZ24/phiEco32 family is highly divergent from the Mx8/Pr family, having split in the early Tailoscene either a little after T7/P22 or as a separate clade from T7+P22. Multiple tubeA lineages were traced to radiation in the early Tailoscene, and have as of yet no representation in any sipho- or myovirus. There may be one or two podovirus-specific portal lineages arising in the early Tailoscene. However, the major head protein appears to be more interchangeable with siphoviruses and myoviruses, and the packaging system is even more so. Thus far, the proposition holds up that to explore ΦGT1 relationships concerning virion structure, assembly, and ejection mechanism to a heavily characterized prototype, P22 would seem to be a promising candidate, although thinking of the LUZ24/phiEco32 lineage, the Mx8/Pr lineage, the T7 lineage, and the P22 lineage as part of unordered ancient radiation is also a good model.

The Mx8/Pr family is not as well populated among sequenced genomes as the LUZ24/phiEco32 family. Although there are numerous *Brucella* phages sequenced, they are all nearly identical. Turner et al. [16] recently published a rationale for removing *Podovirdae* from the official phage taxonomy citing as justification that orthologous core genes could not be established between *Lederbergviruses* (P22) and *Myxoctoviruses* (aka. Mx8), citing a CoreGenes analysis. A relationship between Mx8 and P22 for all the core structural genes is established in this study timing to ancestors ranging from ~2 to 3.5 Gya, with the main ambiguity being whether it is a class of equal depth to P22 of >3 Gya or a subclass of P22 with a major component of core genes related in the 2–3 Gya range. Although we have not included a detailed analysis of the internal virion protein genes here, ΦGT1 IVPB and IVPD find Psi-Blast matches in the syntenic genes of Mx8, leaving little doubt that Mx8 has the classic podoviral structural gene complement. The discrepancy with Turner et al. [16] can be understood as due to the reliance on computational tools that cannot detect similarity in the requisite genes in the 2+ Gya range.

At ~0.7 Gya the LUZ24/phiEco32 lineage recombined with the Mx8/Pr lineage to create the dual tubeA arrangement of the recombinant ΦGT1 family. This recombination event was described in Section 3.2 and timed on each tree in the manuscript and in the Supplemental figures as the node connecting ΦGT1 to the *Sinorhizobium* prophages. The ΦGT1 dual tubeA arrangement descends with little apparent revision of the core structural genes. However, there may well be recombination among recent sibling lineages. Those would not be resolvable within the uncertainty of the tree analysis. The nonstructural gene component among the various ΦGT1 clades was essentially replaced within a mere 0.1 Gya (Section 3.7). Since that degree of churning disrupts establishing congruence with the more coherently descending genes, we do not currently have an applicable method to include them in our analysis.

The junction between the two structural modules is the most unique feature of the ΦGT1-like genome, with two adjacent and highly diverged tubeA genes flanking the junction. That recombination is mapped to a time ~0.7 Gya and has succeeded in spreading into a variety of current host genera. This knowledge can be used to enable a hypothesis-driven approach for further examination of the phage. If one were to choose ΦGT1 for structural studies, for example, one would be armed with the prior knowledge that there is some major structural variation to find, that it involves the tail hub, and that it must be functionally significant to explain the duration and degree of spread of descendants. Since tubeA is a major attachment point for tail fibers and there also appears to be an excess of tail fiber genes in ΦGT1, one would expect to clarify if there is a connection between the unusual tail hub and the number and/or diversity of tail fibers used. One would be alerted that there will either be two rings of the tubeA protein, or one ring of a heterodimer. If there are one or two rings should be clear by even preliminary structural alignment to the ancestral P22 and T7 arrangements. If there was one ring, that would convert the usual 6-fold symmetry of the tail to 3-fold symmetry in approximate 6-fold symmetry. One would be alerted to look for other aspects of 3-fold symmetry imposed on the whole of the tail hub and whether that might impact how the tail fibers transduce the signal through the tubeA ring that the phage is properly positioned for injection. In considering how any altered functionality arose, one could reasonably assume that both ancestors had tail fiber function analogous to T7 or P22 and that the immediate result of the fusion event had to be a functional phage. Recognition of the nature of the dualA arrangement enables other kinds of hypothesis-driven investigation. After being alerted to the possibility of two highly divergent tubeA genes in the same genome, we found another example in the Bcep22-like phages, apparently arising completely independently. Finding this odd recombinant pattern more than once supports the proposition that it has some selective value.

### 4.2. Relationship of the ΦGT1 Family to the Oceanic Metagenome

Liang et al. [2] associate *Sulfitobacter* with the marine family *Roseobacteraceae*. However, although the ΦGT1 genome has multitudinous Psi-Blast matches to metagenomic marine phages, a detailed reconstruction of its history does not include descent as any major phage lineage in *Roseobacteraceae*. The Psi-Blast matches reflect shared ancestry with multiple major marine metagenomic lineages as of the time 1–1.5 Gya when the LUZ24 module entered alphaproteobacteria and distributed them into multiple developing orders. The path to ΦGT1 did not go through those marine phage lineages. Instead, it went through *Rhizobiales* from which it then transferred to *Roseobacteraceae* and made a lineage descending into *Sulfitobacter.* Based on the numbers of close Psi-Blast matches in bacterial and metagenomic databases, that lineage appears to be relatively unproductive, barring some sampling bias that is causing it to be underrepresented. In retrospect, the marine metagenome thus far examined is relatively strongly biased to a narrow slice of oligotrophic planktonic hosts. Marine bacteria found in coastal sediments are a very different niche.

### 4.3. Balance between Horizontal Transfer and Vertical Descent

Incorporation of the many prophages and prophage remnants into the study gives a much more robust accounting of the evolutionary history leading to ΦGT1. This was exploited to explore the balance between the vertical descent of phages from ancestral host taxa versus horizontal transfer across the host range. Since the pivotal review by Hendrix et al. [40] on horizontal transfer and gene reassortment in phages, mentions of vertical descent in phages have virtually disappeared from the phage literature. We make a distinction between gene reassortment and horizontal transfer across host taxa for the following reason. If phage genes only engaged in recombination within a host taxon but never transferred horizontally among them, then every phage gene would ultimately have the same evolutionary history as its host. There could still be confusion based on recombination between paralogous phage lineages within the same host, but without horizontal transfer between host taxa, phage phylogeny would be a much simpler affair. To consider the balance between horizontal transfer and vertical descent, it is necessary to have some confidence in the timing of phage nodes versus host nodes. Quality controls are offered in Figures S1 and S2 to document the degree of success and limitations in our current effort to correlate host and phage phylogenies.

Confinement of phages to vertical descent is obvious on a laboratory time scale and is also reflected in the observation that the most recent phage sequence clusters are mostly, although not completely, confined to a host genus. With the greater time resolution provided by timetrees, we see that the origin of the dual tubeA ΦGT1-like structure module early in *Rhizobiales* is followed by its descendants being concentrated in families and genera of that order. By following the descent of the tubeB gene in that module, we see it passing through the differentiation of families and genera in *Rhizobiales* before transferring to the sister *Rhodobacterales* order to descend into ΦGT1. The simplest explanation would be if phage transmission is also mostly vertical on the 0.5 Gyr time scale, although with enough horizontal transfer mixed in to generate ample confusion. After placing the dual tubeA ancestor in *Rhizobiales* before the generation of the host families, one might expect that all the families and all the genera of *Rhizobiales* would have descendant ΦGT1-like temperate phage lineages; but they do not. Even after accounting for the differences in the numbers of completely sequenced genomes, the success in vertical transmission is remarkably patchy. One genus was found, *Liberibacter*, where there are ΦGT1-like copies, but all copies found are full of nonsense codons. So ΦGT1 phages in *Liberibacter* appear to be in the process of failing. Patchy distribution in vertical descent requires that there be at least soft boundaries to horizontal transfer that develop as bacterial taxa differentiate.

Mixed with a history of patchy vertical descent there are clear examples of horizontal transfer, meaning that the node relating to the phage genes is significantly younger than the node relating to the hosts that they occupy. For example, dual tubeA ΦGT1-like genomes in *Rhizobiales* and *Rhodobacterales* are related at ~0.7 Gya. Those host orders diverged by >1.6 Gya according to the TimeTree website. Hence that represents a horizontal transfer when the hosts were about as diverged as bacterial families are today. The dual tubeA module is well represented in *Rhizobiales*, both in total numbers and numbers of genera and species occupied. However, in *Rhodobacterales* it is a minor lineage and found only in the family *Roseobacteraceae*. We consider it most likely, based on a simple mass-action argument that the source of the transfers was the more heavily populated host taxon. The entire core structural module appears to have been transferred intact. Similarly, we found that the dual tubeA genome made a transfer to form a very small clade back in gammaproteobacteria confined to the genus *Vibrio*. We could find no evidence that any of the other component genes of the phage succeeded in making the transient together with the core genes, either in the homologous prophages or scattered into any other sequenced prophages in the recipient host taxa. That could mean either that only part of the phage genome was transferred, or that only part of the phage genome had sufficient selective value to be retained and amplified after it was transferred.

For horizontal transfers occurring in the last 0.5 Gya, it seems typically true that the recipient side of the transfer is lightly populated. Another example is *Sinorhizobium* phage ΦM5. We initially thought that since the LUZ24 donor to make the dual tubeA genome was apparently in early *Rhizobiales* that this descendant LUZ24-like phage was another example of vertical descent within *Rhizobiales*. However, although ΦM5 is a temperate phage of *Sinorhizobium*, we found no relative prophages of it in sequenced *Sinorhizobium* genomes, and only one prospective prophage anywhere else in *Rhizobiales*. When metagenomic sequence was added, it became very clear that at least the early descent of ΦM5 occurred in the marine bacterial SAR116 taxon (Section 3.8), which upon examination by Lee et al. [41] would appear to be a separate alphaproteobacterial order. It would not be surprising if other clean lines of vertical descent were discovered upon collection of additional sequences to have detoured through some remote host taxon.

The earliest horizontal transfers we have characterized are of the LUZ24 module between gamma- and alphaproteobacteria, and of the Mx8 module between delta and alphaproteobacteria. In each case, the transfer is well after the differentiation of the host classes, but we have no evidence at this time to conclude in which host classes the modules originated. Another source of ambiguity about horizontal transfers is that if the transfer is soon after the host differentiation, the relative timing of the nodes will not be different enough to be distinguishable. Since some models would suggest that recently related hosts are much more likely to exchange DNA, that effect could cause the timetrees to gloss over considerable chaos.

One way to clarify the ambiguities of measuring both horizontal transfer and vertical descent is to propose that the load of phage lineages is stationary (meaning an observer at any point in time would see approximately the same load of phage lineages per host taxon). Since there is some horizontal acquisition of phage lineages, a balancing loss of phage lineages is nearly a theoretical necessity. Otherwise, over long evolutionary time, the predicted diversity of phage lineages per taxon would be predicted to continuously increase, possibly even exponentially. Incorporating a balance between lineages gained and lineages lost would avoid those sorts of instabilities and might provide a useful guide for sorting out the observational data.

### 4.4. Onset of the Host Range Barrier

On the low time zone end of the vertical vs. horizontal transmission problem, we looked at more detail at the *Sinorhizobium* “m” vs. “f” lineages. In light of the considerations above, a soft barrier to horizontal transfer does have to at some time arise between diverging host taxa. It would have to be strong enough to explain the frequent observation that one descendant bacterial family or genus vertically inherits a phage lineage, and a sibling host taxon does not. Taking the diversification between *S. meliloti* and *S. fredii* as an example of a phage vertically transiting a host diversification, we note that the present TimeTree website estimation of the *meliloti*/*fredii* split is 67 Mya, in good agreement with our best estimate of the differentiation of the “m” and “f” lineages at ~77 Mya, with “m” prophages dominating in *S. meliloti* and “f” prophages dominating in *S. fredii* (Section 3.7). The criteria used by taxonomists to declare different bacterial species are many and always in a state of flux, but the agreement between these two numbers suggests that the consensus of criteria about what makes these different species of bacteria has matched the onset of a barrier that would allow the vertically descending phage lineage to differentiate into *meliloti* and *fredii* branches. However, when the analysis was extended to a collection of 20 “m” and “f” prophages, three instances of a bacterial strain containing one each of them were found. This would seem to suggest that at its inception, the boundary is much leakier than it will eventually become.

## 5. Conclusions

This study provides insights into the evolutionary history of the lytic podovirus ΦGT1 and its core structure module. Using carefully curated alignments and timetrees, the research traced the origin of the ΦGT1 core structure genes through a series of common ancestors with a variety of classified and unclassified podoviruses including lytic and temperate families back to a radiation of the podoviral form close to the origin of life on Earth. One major recombination between two distinct lineages produced the unusual dual tubular tail A arrangement of the ΦGT1 family early in the development of the alphaproteobacterial order *Rhizobiales.* The study detailed the phage’s evolution as marked by both vertical transmission with patchy persistence and horizontal transfer with respect to host taxonomy. Overall, this research provides a unified framework for understanding the evolution of podoviruses.

## Data Availability

Alignments and HMM models corresponding to each of the trees reported are available at https://Stephen-Hardies.github.io (accessed on 10 May 2023).

## Supplementary Materials

The following supporting information can be downloaded at: https://www.mdpi.com/article/10.3390/v15071475/s1, Figures S1 and S2: Controls on timetree quality; Figures S3–S12: Timetrees for each ΦGT1 core structure and morphogenesis protein; Table S1: Node times used to calibrate timetrees; Table S2: Prospective prophages used in the study, and replacement/synonymous analysis of selective pressure in the *Sinorhizobium* prophage lineages; Table S3: Mass spectrometry data.
