# Supplementary material for: Identification of Structural and Morphogenesis Genes of Sulfitobacter Phage ΦGT1 and Placement within the Evolutionary History of the Podoviruses
Source: Viruses. 2023 Jun 29;15(7):1475. doi: 10.3390/v15071475 (PMC10386132; doi:10.3390/v15071475)
Supplement: Supplementary file 1 [file viruses-15-01475-s001.zip › viruses-2419898-supplementary.pdf]

Supplemental Figures

Figures S1 and S2 are part of a set of controls examining the quality of the time scale measurements

Single gene versus the phylogenomic approach

Figure S1 is a single gene tree using protein sequences from just one of the bacterial core genes (IF-2). The question is whether the 95% Height Posterior Density (HPD) intervals from a single gene are of similar width and median node heights as concatenated phylogenomic constructs.

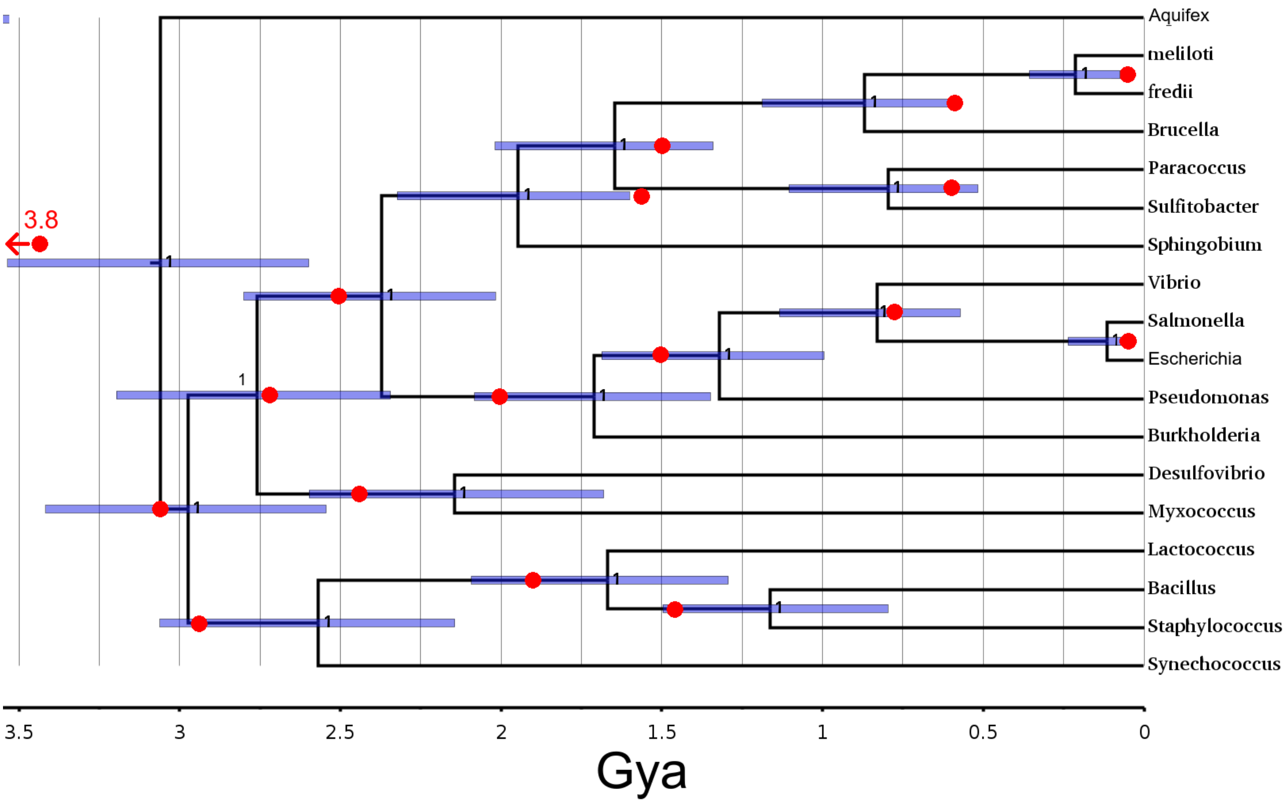

Figure S1 Recapitulation of the bacterial phylogenomic tree from IF-2 sequences alone. The selection of taxa was arbitrary, and intended to cover a wide range of close to distant relationships. The tree was made as described for the phage genes in methods. Red dots indicate the split times from the TimeTree web site (http://timetree.org). The time scale was calibrated to minimize deviations from the TimeTree web site values excluding Aquifex. Blue bars indicate 95% HPD intervals.

The single gene performance mostly recapitulates the phylogenomic result. The widths of the 95% HPD intervals are similar to the phylogenomic tree. Most splits estimated by the phylogenomic method fall within the 95% HPD of the single gene tree. We would conclude that the one point where the phylogenomic time is well outside the calculated 95% HPD (Aquifex) probably indicates a horizontal exchange affecting that taxon.

In a thoroughly done phylogenomics study such as Battistuzzi et al. (2004), the prospective core genes are screened first for approximate congruence. The protein sequences are then concatenated to become the substrate for the tree analysis. Individual deviations such as the Aquifex case above are assumed to only influence a small portion of the alignment columns in the concatenated sequence, thus having minimal influence on the outcome. Those circumstances don't pertain to phage genes, so we don't do the concatenation.

Bayesian median node heights versus maximum likelihood node heights.

We have noticed that Bayesian median node heights seem suspiciously high for closely related sequences. On Fig. S1, note that the TimeTree result falls to the bottom of the 95% HPD intervals for nodes <= 0.5 Gya. A control was incorporated in which duplicate sequences were added to the tree. Since the unscaled node heights are in expected divergence, the node height connecting duplicate sequences should correspond to < 1 difference per the length of the sequence. However, the median Bayesian node heights typically came out 5 time or more larger than that. The same trees evaluated by maximum likelihood adhered to the lower expectation. This makes some sense, since the Bayesian estimates are medians of trees that are close to but not quite maximum likelihood. If the truth is close to zero, then almost true will tend to be systematically high.

Residual saturation effect.

We noticed that the genes selected for bacterial phylogenomics tend to be far less diverged and more easily alignable across the root of life than are phage genes. For phage genes, we routinely required position-specific matching profiles in the form of a Hidden Markov Matrix (HMM) to detect similarity and effect alignment across the most distantly related clades. We thought it could be true that the similarity across the most distantly related clades might be saturated under the more rudimentary BLOSUM matching operation used by MrBayes, and wondered what distance would it produce under saturation. To evaluate this problem, a random sequence was introduced into each alignment to see where MrBayes places sequences that are completely saturated. In the IF-2 control, the random sequence was detected as a much longer branch (not shown). But for the phage gene trees, the random sequence joined as an equal length branch within the midpoint 95% HPD interval. Under these circumstances we believe that the distance extrapolation ceases to be linear with time but instead converges on some arbitrary value representing the degree of similarity expected to random sequence. Most of the phage gene trees are calibrated in the lower time ranges, and then extrapolate back to 2.5 - 3.5 Gya for their most ancient nodes. For example, tubeB (including T7 from Hardies et al. (2016)) extrapolates to ~3.2 Gya, and tubeA (fig.2) extrapolates to ~ 2.4 Gya. Given the saturation effect, both may have arisen by 3.2 Gya, or even at the root of life at 3.8 Gya.

Quality of the large terminase tree for calibration purposes

The large terminase tree was calibrated differently. Its root was placed at 3.8 Gya. If there is curvature in due to saturation in that tree, it will make all of the nodes seem older than they really are. And since the large terminase tree was used to calibrate all others, that effect would be conferred upon the entire analysis.

To get a handle on how much saturation has perturbed the large terminase tree, we used it to calibrate one tree where there is an outside benchmark. The T7 single chain RNA polymerase is homologous to mitochondrial RNA polymerase (Cermakian et al., 1997) through the mitochondrial endosymbiosis currently thought to be ~1.9 Gya (Wang and Luo, 2021). So we calibrated the RNA polymerase tree against terminase, adding mitochondrial RNA polymerases, and DNA pol I as an outgroup. The pol I outgroup provides some protection against unequal rates on the eukaryotic and prokaryotic sides of the tree.

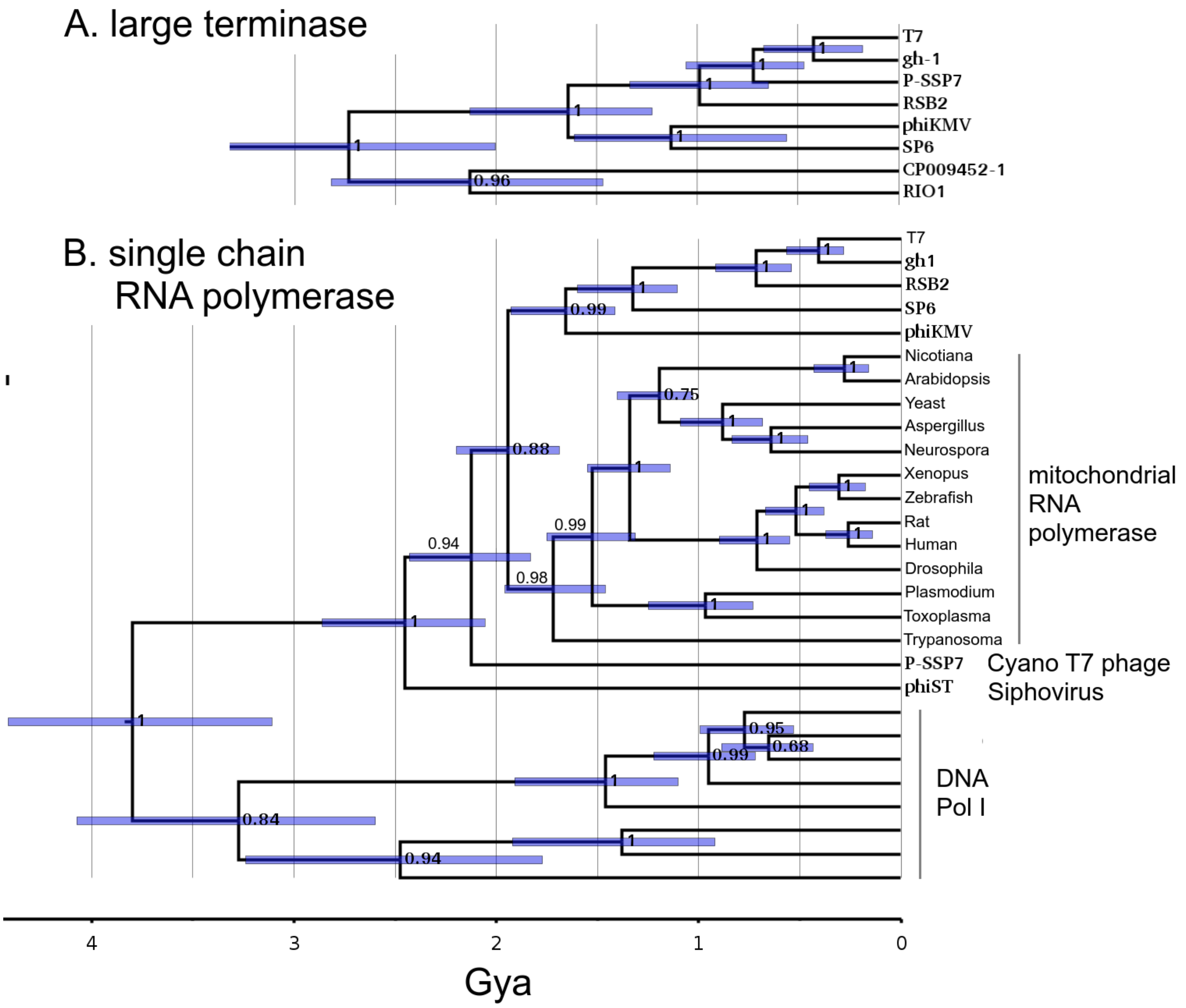

Figure S2 T7 RNA polymerase timetree including connection to mitochondrial RNA polymerase as scaled based on the large terminase tree. All of the included mitochondrial RNA polymerases are nuclearly encoded. The segment used for tree construction was limited to the portions of RNA polymerase and DNA pol I exhibiting structural homology. Nodes used for calibration were T7/gh-

1 and T7/phiKMV. Other nodes are roughly consistent within error, except the cyanophages exhibit a clear recombinant pattern of descent of the RNA polymerase relative to the large terminase. The root position of DNA pol I versus RNA polymerase was not set to 3.8 Gya, but rather extrapolated to that position based on the scaling relative to the large terminase tree.

Figure S2 reveals that scaling of the RNA polymerase tree relative to the terminase tree places the mitochondrial endosymbiosis close to 1.9 Gya and the root of the RNA and DNA polymerases near the beginning of life. The reader should beware that different selections of sequences to include and nodes for calibration may move the median node heights within the indicated uncertainty intervals. It isn't normally expected for calculated node heights to fall right on a prediction as they have in this case. The large terminase time scale does not appear to be distorted by saturation in this test, although, given the uncertainty intervals a compression on the order of 0.5 Gya could not be excluded. A version of the tree without the clock specification did not show a systematic difference in rate between eukaryotic nuclearly encoded RNA polymerase and phage encoded RNA polymerase. However, mitochondrially encoded RNA polymerase and the N4-like (Schitoviridae) phage virion RNA polymerase family both were detected by that control to evolve faster, and were hence excluded from the timetree analysis. The N4-like virion RNA polymerases also joined the tree in the 2-2.5 Gya range.

The rooting of the large terminase tree at the beginning of life was initially based on the premise that phages were involved in the invention of DNA and the transition from RNA- to DNA-world. The fitting of the mitochondrial RNA polymerase transfer to the mitochondrial endosymbiosis provides some objective support for this time scale.

The following are timetrees as seen in the main text with additional technical information, and timetrees for additional core structural genes of ϕGT1.

Large Terminase subunit timetree

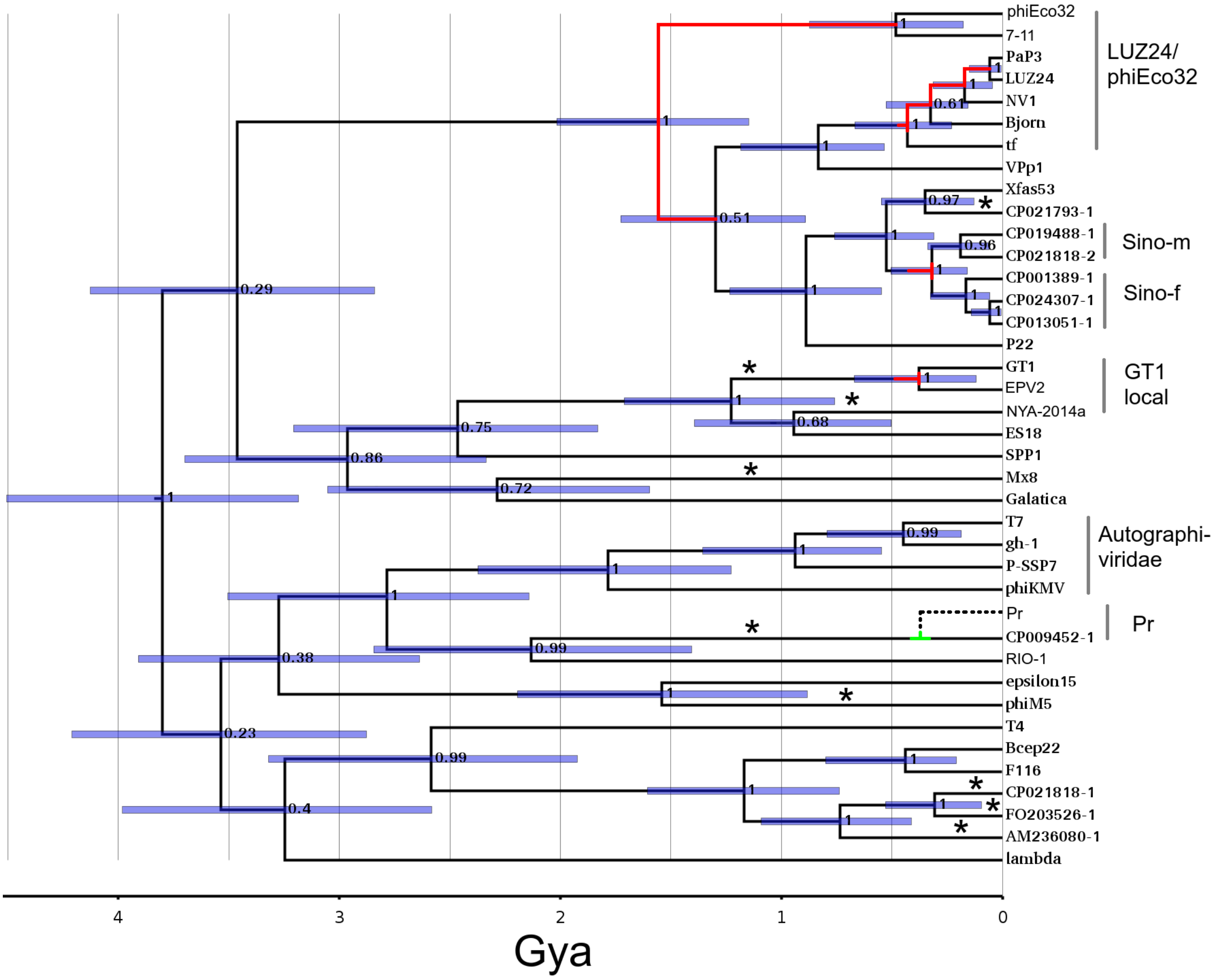

Figure S3 Large terminase subunit P-loop domain timetree with ϕGT1, selected close homologs, and selected other phages covering the breadth of tailed phage tree space. This is a subset of the global terminase tree first reported in Serwer et al. (2004). That tree encompassed all reported large terminase sequences at the time, and we have continued to expand it finding no examples of a phage without a large terminase gene that can be added to that global alignment (including the phi29-like packaging ATPases). Whereas the global terminase alignment covers both domains of the large terminase, this tree covers only the P-loop domain to avoid several cases in which the C-terminal domain was found to be replaced by recombination. This tree was scaled by placing the root at 3.8 Gya. Nodes indicated in red or green maintain congruency with the other structural genes of the LUZ24, or Mx8/Pr modules, respectively.

The ϕGT1 large terminase clade itself is especially chaotic, and its details were mostly sorted out after the outline of the less convoluted terminase clades was established. ϕGT1 has an intragenetic recombinant large terminase with the N-terminal domain related to prototype ES18 as shown, but the C-terminal domain related to the F116 terminase lineage. The recombinant form tracks back to just after the common ancestor with ES18 at ~ 1.25 Gya, and is shared by *Pseudomonas* phages

LKA5 and H66 in gammaproteobacteria where the recombinant presumably formed. EPV2 contains that recombinant form marking that it had arrived in alphaproteobacteria by ~ 0.4 Gya. The *Sulfitobacter* phages pCB2047-A, pCB2047C, and NYA-2014a, which for most genes in the structure/morphogenesis module are very close to ϕGT1, also have an ES18/F116 recombinant large terminase subunit structure. But theirs is a different recombinant with both domains having a different history than those ending up in ϕGT1 and EPV2. Large terminases or fragments of large terminase related to the F116 lineage are also found sporadically in other parts of the alphaproteobacterial collection examined here. Another oddity of the ϕGT1 large terminase clade is that it is composed of a mixture of the different morphotypes (e.g., ES18 is a siphovirus). The large T7 and P22 large terminase clades do not (recognized as of yet) have verifiable non-podovirus members of < 2-3 Gya common ancestry.

The P22 large terminase itself is descended from the LUZ24 common ancestor. The close association of P22 and Xfas53 large terminases with LUZ24 and alphaproteobacterial sequences is not characteristic of other P22 genes. This indicates that the increased tendency for recombination of the large terminase gene over structural genes extends to horizontal transfer across large phylogenetic distances. The *Brucella* phage Pr terminase is shown by a dotted line because it has an even more strange recombinant structure that requires a separate tree build to locate it. All of the *Brucella* phages carry an intein within the N-terminal P-loop domain. Downstream of the intein, the sequence maps as shown above. Upstream of the intein the sequence has completely different ancestry, more closely related to ES18. CP009452-1 is a prospective prophage which is the closest sequence we could find with the parental non-recombinant large terminase sequence and it contains the entire Mx8/Pr structural module. However CP009452-1 is from genus *Sphingophyxis* and is representative of a small family in alphaproteobacterial order Sphingomonadales. There are other related sequences in a variety of families of Rhizobiales not much further away, and most members of the Mx8/Pr lineage are in Rhizobiales. So, if one is parsimonious about the number of transfers across host orders, the common ancestor existed in Rhizobiales at ~ 0.45 Gya and happened to reveal itself by a transferant thrown off into Sphingomonadales and a recombinant to produce a lytic phage in *Brucella* without leaving any traces of prophages in that genus.

Portal timetree

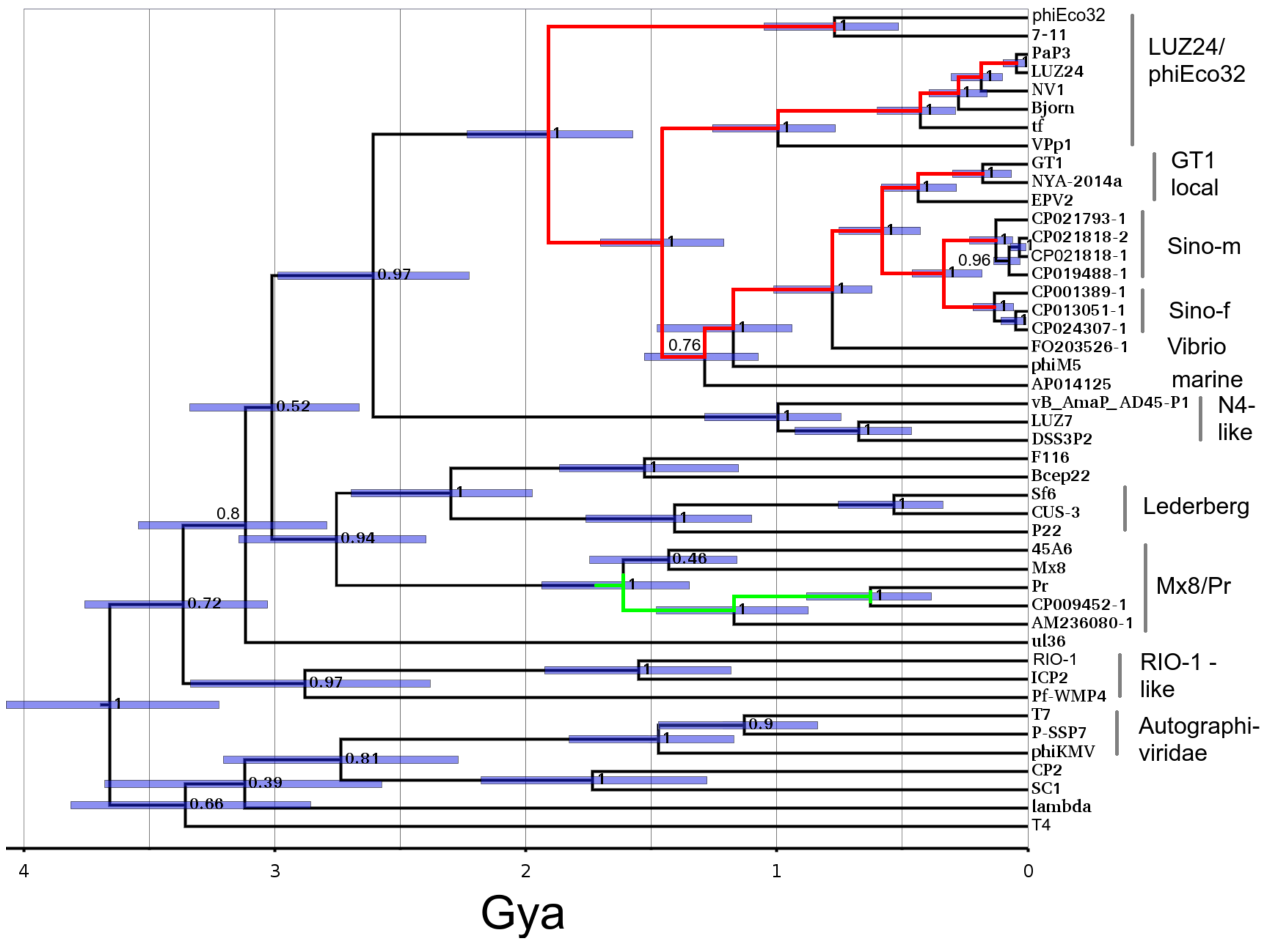

Figure S4. Timetree of ϕGT1 portal protein with selected close homologs and other selected phages to fill out the tailed phage tree space. Calibration was by best fitting to average node heights from Tables S1A and S1B as well as Pr/CP009452-1 and T7/P-SSP7 from fig. S3. Nodes in congruency with the LUZ24 module in general are indicated in red. A set of nodes that are in general congruency throughout the Mx8/Pr structural module are in green. In the large terminase tree, Mx8 and AM236080-1 are not clustered with Pr and CP009452-1, but throughout the structural genes they have the same topology as the portal tree. This is part of a ~ 2000 member portal alignment which contains representatives of most but not all phage groups at this time. Quality of alignment judged by HHpred HMM to HMM alignment between the various clades indicated that quality aligned regions are dispersed across the alignment within the clades for ϕGT1, LUZ24, P22 , and Mx8, but become localized to just a region or two for the relationship between this larger P22-like group and the T7, lambda, and T4 groups. Because there is no large single contiguous region of good alignment to collapse the calculation around, the tree was made with the full length SAM alignment.

Although N4-like (aka. Schitoviridae and related phages) podoviral proteins in general are difficult to associate with the other podoviral classes, interestingly the N4-like portal joins high on the LUZ24/ϕGT1 clade. This could be explored as sequence marker of podoviruses for prophages and metagenomic sequences that don't have a tubeB gene. The T7 clade, including the podoviruses DC1 and CP2, is as far removed from P22 as it could be, which is part of the support for a most ancient split between P22 and T7 subclasses of podoviruses. The ambiguity window at the root

obscures whether T7 and P22 could be a single pan-podoviral clade, with the strongest challenge to that being ul36 (representing the Lactococcal siphoviruses) joining high on the P22-like clade.

ϕGT1 gp6 timetree

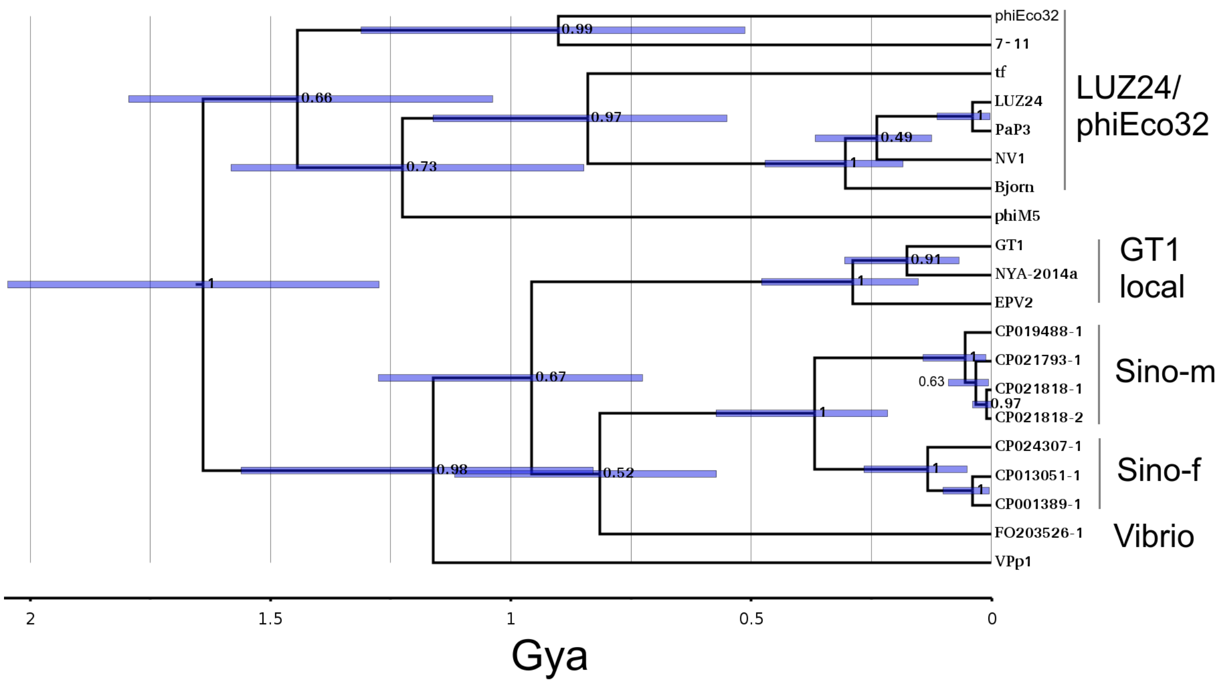

Figure S5. Timetree of ϕGT1 gp6 and selected homologs. ϕGT1 gp6 is a 75 residue protein encoded prior to the putative scaffold protein. Calibration was the best fit to the average node heights of those nodes that appear in Tables S1A and S1B, except the phiM5 node was not used for calibration because it appears to be ambiguously located relative to the midpoint. The LUZ24 homolog is gp64; The VPp1 homolog is gp27, which was not recognized until the frame was extended to an upstream start codon over that annotated in GenBank. Targeted searching was required to find the more divergent homologs on this tree. Targeted screening of metagenomic sequences reveals that this protein is also a characteristic of the LUZ24-like marine metagenome clades.

Scaffold timetree

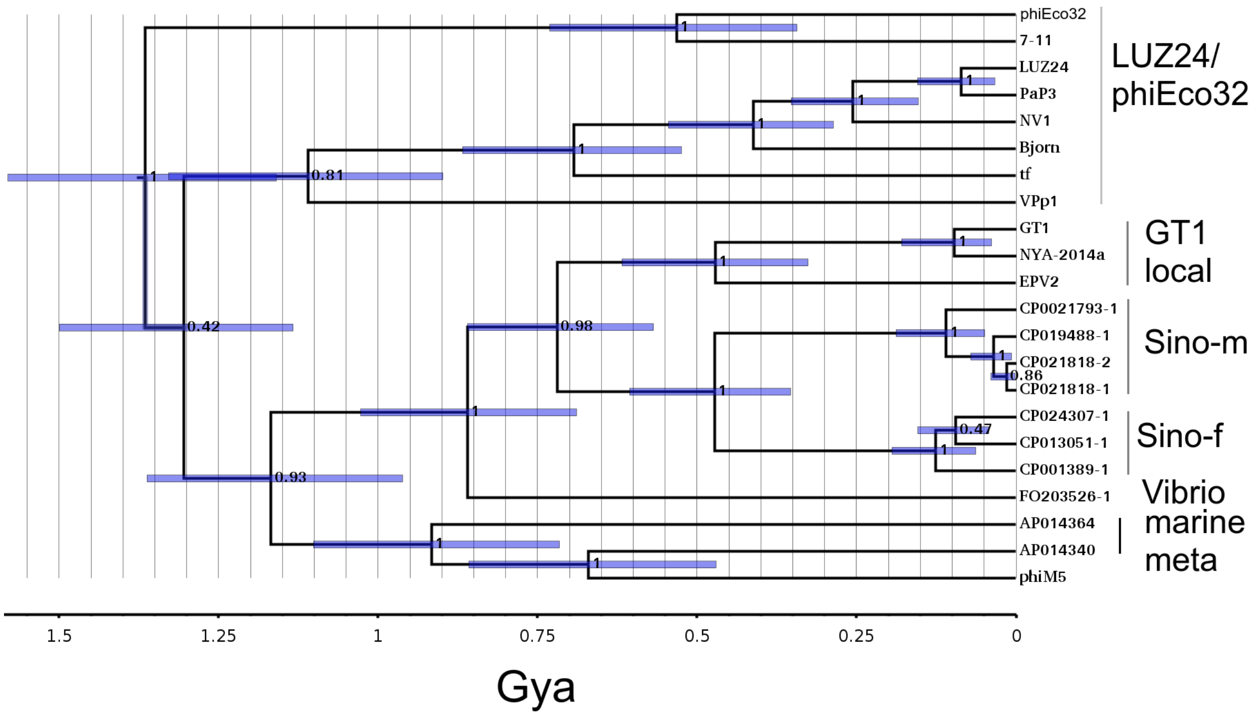

Figure S6. Timetree of ϕGT1 scaffold and selected homologs. Calibration was by best fit to the average node heights tabulated in Tables S1A and S1B. A significant HMM to HMM match was established to the P22 scaffold, but we considered the quality of alignment as scored by HHpred to be too low to attempt to include the P22 clade in the MrBayes tree.

Major Capsid Protein timetree

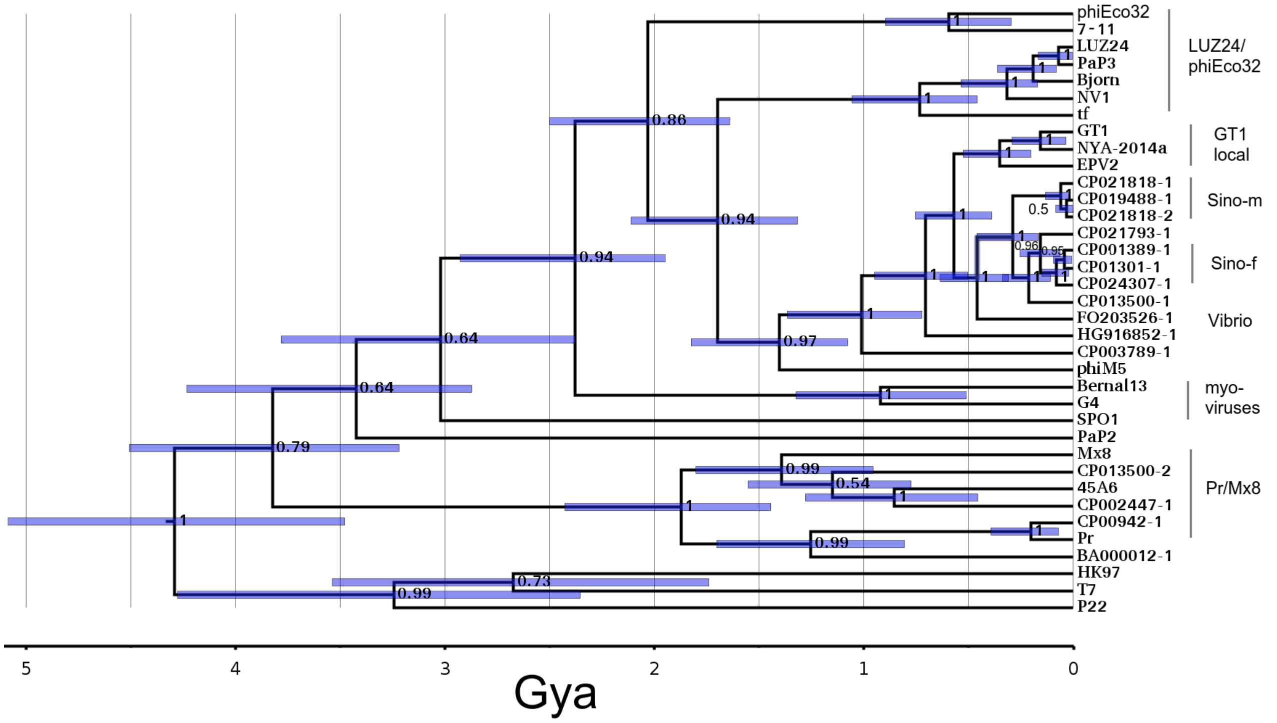

Figure S7. Timetree of ϕGT1 major capsid protein with selected homologs of various depths of divergence. Calibration was with the same set of nodes as was used for portal. This tree is compatible with that shown by Johnson et al. (2017) to illustrate the relationship of phiM5 with LUZ24, except for some branch swapping in places where their tree reported poor topological support. We attribute their lesser resolution to alignment difficulty. The SAM aligner rejected attempting alignment across the sequences joining above 3.5 Gya. Those sequences were added by making separate HMMs for each of them and then adding them to the ϕGT1 family alignment by enforcing the HMM to HMM alignment given by HHpred. Limiting the characters sent to MrBayes to those with good posterior residue alignment scores by HHpred among ϕGT1, T7, HK97, and P22 reduced the number of usable characters to 120, but didn't improve the definition in the upper time zone. This is presumably because the similarity in that range is too weak to be measured by the MrBayes distance metric. Hence we allowed MrBayes to process the full alignment to get the maximum definition in the lower time zones. Basically, all one can tell from the top of the tree is that HK97, T7, and P22 are ambiguously mixed into the root position. It is apparent that there is not a universal podoviral major capsid protein in general, but the closest verified homologous non-podoviruses to ϕGT1 that we found are the myoviruses marked at ~ 2.4 Gya.

ϕGT1 gp10 timetree

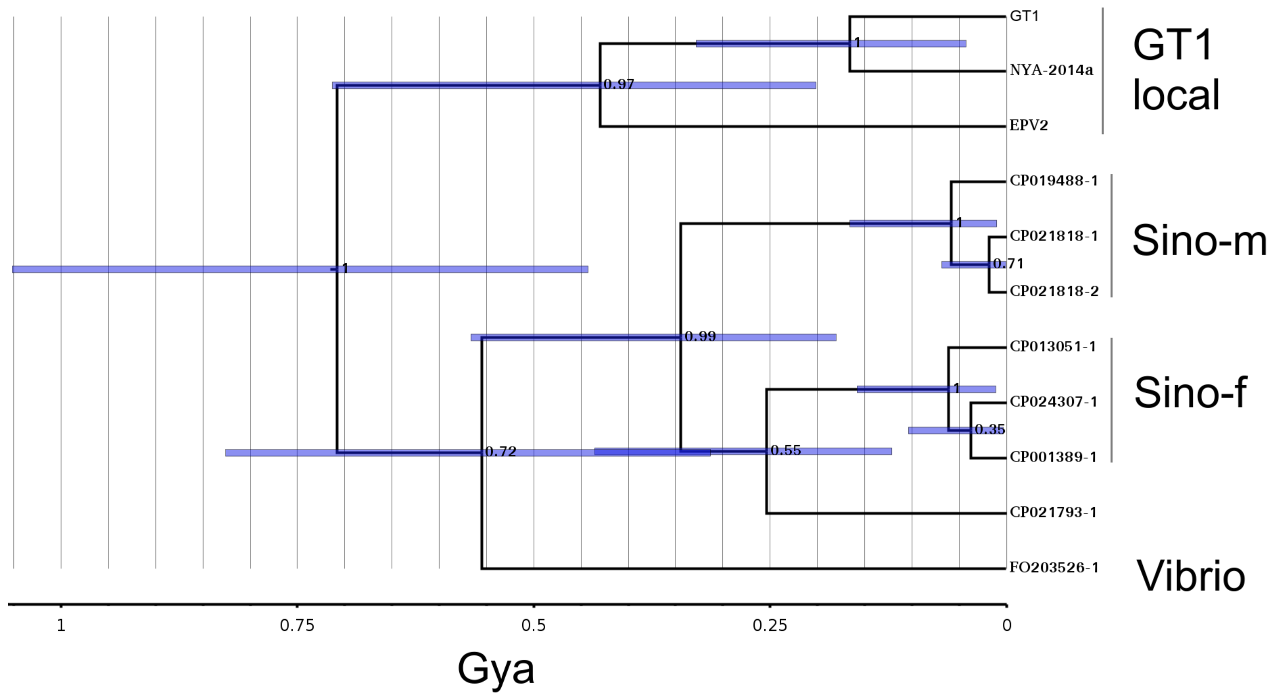

Figure S8. Timetree of ϕGT1 gp10 and selected homologs. Those nodes that are included in Table S1B were used for calibration. ϕGT1 gp10 is a 52 residue protein found conserved between the head and tail modules of the ϕGT1-like dual tubeA clade, but has not been traced any deeper in time. Significant matching across the midpoint required targeted searching.

ϕGT1 gp11 timetree

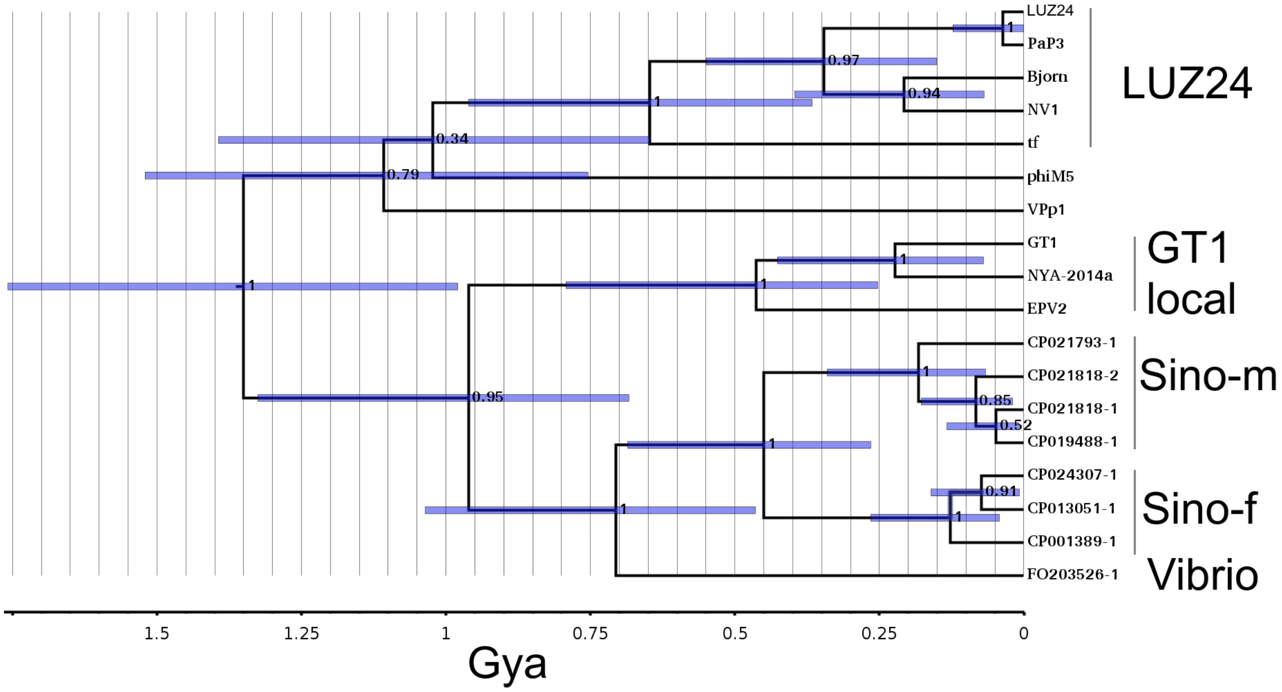

Figure S9. Timetree of ϕGT1 gp11 and selected homologs. Those nodes that are included in Tables S1A and S1B were used for calibration, except phiM5 was excluded because it is ambiguously located relative to the midpoint. ϕGT1 gp11 is a 107 residue protein encoded between head and tail modules, and has been detected to be in the mature ϕGT1 virion by mass spectrometry.

Tubular tail A timetree

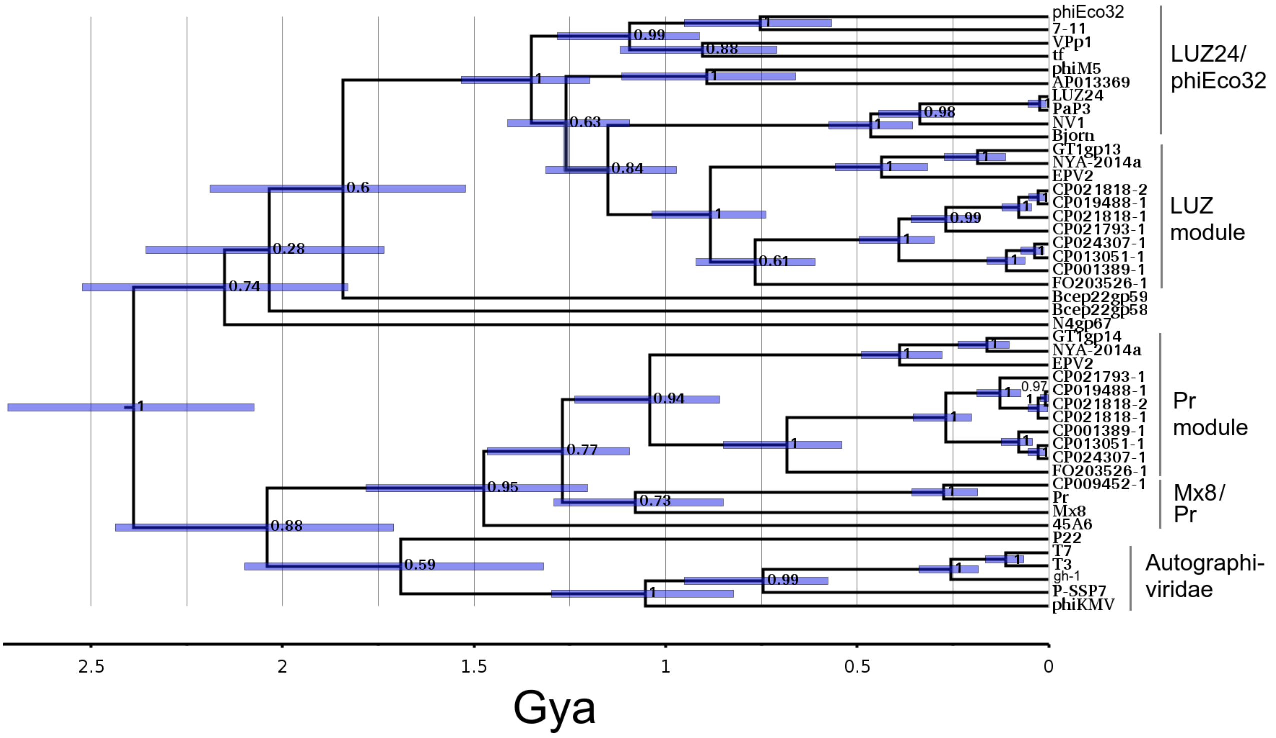

Figure S10. Timetree of ϕGT1 gp13 and gp14, the LUZ24-like and Mx8/Pr-like tubular tail A paralogs and selected homologs. Calibration used the usual nodes from Tables S1A and S1B, except tf/LUZ24 was excluded since tf has been moved to a recombinant position. It also used all the nodes in the dual tubeA part of Table S1A twice: once for the gp13 clade and again for the gp14 clade. Also used were Pr/CP009452-1 and T7/P-SSP7 from figure S3. Although SAM produced an alignment that captured essentially the same tree, the alignment was reworked across all the deep nodes using HHpred HMM to HMM comparisons to increase the numbers of correctly aligned residues. Because variant tubeB lineages tend to merge at ~ 3 Gya, we suspect that the top of this tree may be compressed by a saturation effect.

Tubular tail B timetree

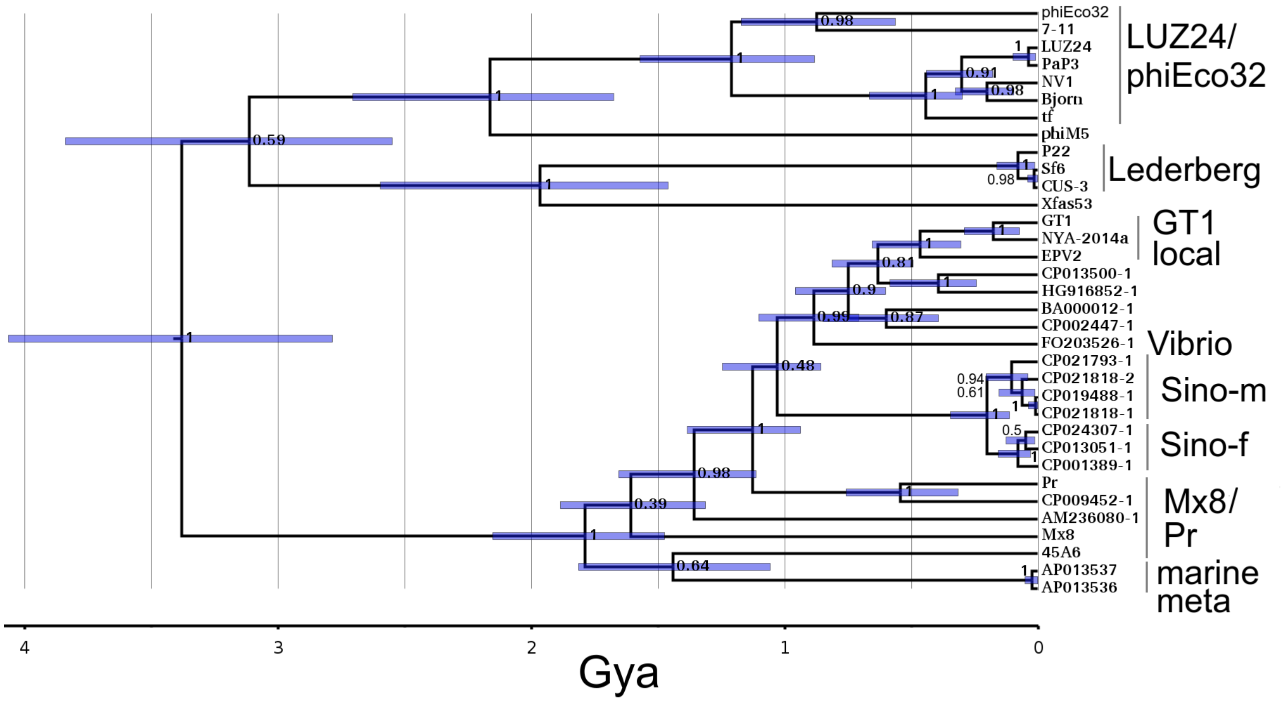

Figure S11. Timetree of ϕGT1 tubular tail B and selected homologs. Calibration nodes from Tables S1A and S1B were used except for phiM5 and NV1, which have clearly been altered by gene reassortment, and in addition Pr/CP009452-1 from figure S3 has been used. There are few marine metagenome matches to flesh out the Mx8/Pr clade, but two are indicated. The alignment of the upper viruses in each clade, phiM5, 45A6, and Mx8, were subjected to realignment based on an HMM to HMM comparison using HHpred. The region used consists of residues 169 to 394 in ϕGT1, which corresponds to residues 358 to 553 in T7 tubeB gp 15.

ϕGT1 gp16 timetree

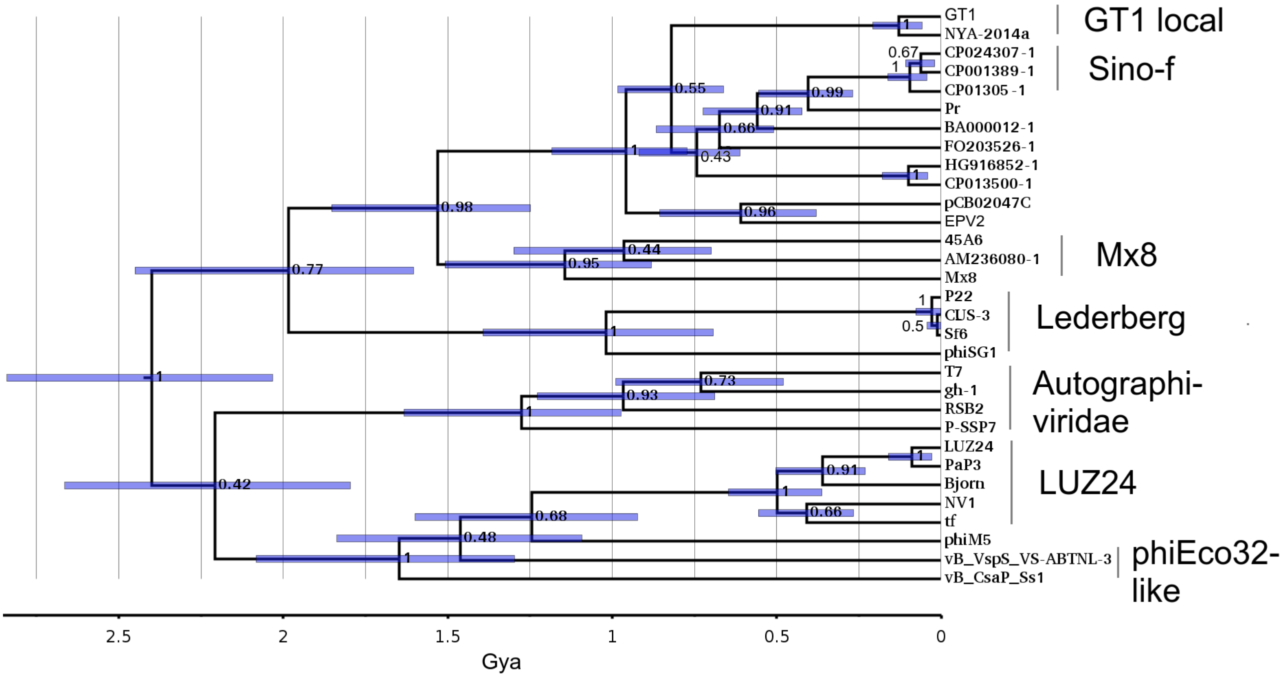

Figure S12. Timetree of ϕGT1 gp16. This gene is homologous to P22 gene 14 and more distantly to T7 IVPA. It has similarity to multiple cellular acetyltransferase families by HHPred. The gene, when present, is invariably at the head of the internal virion protein module, but the protein is generally not found in the virion by mass spectroscopy. It is a required assembly protein in P22. Numbers of phages have a different protein in the syntenic position that does not have acetyltransferase similarity. The Sino "m" clade and phiEco32 itself do not have a homolog of this protein, although a couple of other phiEco32-like phages that do have it have been added to the tree. Nodes that could not be used for calibration are phiM5, EPV2, the Sino-m clade, phiEco32, and Pr. AM236080-1/Mx8 was added from Fig. S11 and all of the nodes in the T7 clade were added based on timing on a separate Autographiviridae tree to flesh out the root position. When representatives of the bacterial pdb:5ISV, 3F8K, 4J3G, 4RI1, 4MI4, and eukaryotic 5NNP families were added to the tree without a clock, they joined at the root position, but diverged at 1/2 to 1/3 the rate of the phage families. Therefore, they could not be included in the timetree calculation. However, by joining at the root, they suggest that either the root is really at 3.8 Gya and the tree is suffering from saturation, or that acetyltransferases were not invented until the time of the mitochondrial endosymbiosis at ~ 2.0 Gya. Since we think that the latter is unlikely, we suspect that the top of this tree is compressed by saturation and the root is really at ~3.8 Gya. All of the cellular families use acetyl-CoA as an acetyl group donor and acetylate widely varied substrates. HHpred HMM to HMM matching indicates that quality alignment of the phage genes is focused to the acetyl-CoA binding site.

Table S1A. General congruence of structural genes in selected LUZ24-related phages.

		LUZ24: phiEco32	LUZ24: tf	LUZ24: Bjorn	LUZ24: NV1	LUZ24: PaP3[b]	phiEco32: 7-11
gp66	L. terminase	1.56	0.57	0.74	0.37	0.01	0.66
gp65	portal	1.56*	0.38	0.27	0.19	0.04	0.73
gp64		1.56*	0.87	0.34	0.27	0.04	1.05
gp63	scaffold	1.56*	0.93	0.61	0.51	0.11	0.45
gp62	MCP	1.56*	0.58	0.17[a]	0.25	0.06	0.48
gp61			0.77*	0.29	0.21	0.03	
gp60	tubeA	1.56*	1.28	0.59	0.46	0.03	1.06
Ave			0.77	0.45	0.34	0.05	0.74
	tubeB	1.56*	0.68	0.43	0.33	0.06	0.37

*The indicated median node heights are in Gya taken from an initial calibrated time tree for each gene, with the calibration point pinned to the large terminase gree at the node indicated by an asterisk. The general consistency of the progression of nodes for each gene was characteristic of the high degree of congruency seen among trees of the structural genes of the lytic LUZ24-related phage group. For use in fine tuning the calibration of the final trees a best fit to the average of available calibrated node heights was used, thus reducing the effect when the initial LUZ24/phiEco32 calibration point was particularly high or low within its uncertainty interval. The best fit was calculated as described in methods section 2.7. Although the phiGT1-related phages did not have the LUZ24-like tubeB variant, within the LUZ24-related phages themselves congruity continued into the tail module as indicated by the tubeB entry above. The rows for tubeB and gp61 were not included in the averages calculated.

[a]Bjorn having moved inside NV1 is supported with posterior support ~= 1. This is one of the few cases of an apparent gene reassortment with the LUZ24 core structural genes.

[b]PaP3 is included as a lytic phage in this cluster although it was originally reported to be temperate. That assignment was questioned by Ceyssens et al. (2008), and we could find no instances of a PaP3-like gene in any of the numerous fully sequenced *Pseudomonas* genomes.

Table S1B. General congruity of the LUZ24 module in phiGT1-related phages

		GT1: phiM5	GT1: *Sinorhizobium*	m/f: FO203526-1	GT1:EPV2	GT1:NYA-2014a	m:f
gp2	Lg. terminase				0.38		0.32
gp3	portal	1.19	0.47	0.63	0.36	0.15	0.27
gp6		1.56	0.92	0.78	0.28	0.17	0.36
gp7	scaffold	1.18	0.73*	0.86	0.48	0.10	0.46
gp8	MCP	1.06	0.47	0.39	0.26	0.13	0.23
gp10			0.73*	0.58	0.44	0.17	0.35
gp11		1.03	0.73*	0.53	0.35	0.21	0.44
gp13	tubeA_L	1.56	1.04	0.89	0.51	0.15	0.31
average		1.34	0.73	0.67	0.36	0.15	0.32

*The indicated median node heights are in Gya taken from a timetree with the LUZ24/phiEco32 node, if available for that gene, set to 1.56. For genes without a LUZ24/phiEco32 node, the average of 0.73 Gya for the phiGT1:*Sinorhizobium* m and f clades was used for preliminary tree calibration, and indicated by an asterisk. The rows with asterisks are present only to show the achievable consistency. Only the node heights based on LUZ24/phiEco32 were used to calculate the averages subsequently used to fine tune the individual time trees as indicated as described in methods section 2.7.

Table S2A. Prospective prophages used in the study.

Name[a]	Start[b]	Stop	Bacterial taxonomy	Phage taxonomy	Length att site	Uninserted chromosome
CP019488-1	539503	577833	*Sinorhizobium meliloti*	Sino m	57	CP021804
CP021793-1	921202	963806	*Sinorhizobium meliloti*	Sino m	56	CP019584[c]
CP021818-1	3167662	3129037	*Sinorhizobium meliloti*	Sino m	12	CP009144
CP021818-2	3519875	3482679	*Sinorhizobium meliloti*	Sino m	45	CP021804
CP013051-1	2169547	2132870	*Sinorhizobium americanum*	Sino f	nd[d]	
CP001398-1	1977689	1934985	*Sinorhizobium fredii*	Sino f	nd[d]	
CP024307-1	2342271	2286585	*Sinorhizobium fredii*	Sino f	nd[d]	
CP009452-1	972415	928774	*Spningopyxis sp.*	Pr lineage	48	CP013344
AM236080-1	4180480	4141988	*Rhizobium leguminosarum*	Pr lineage	14	CP001622
FO203526-1	1699934	1739682	*Vibrio nigripulchritudo*	Transferred to *Vibrio*	57	CP016095
CP013500-1	1068305	1112550	*Rhizobium esperanzae*	Other clade in Rhizobiaceae	49	CP013595
HG916852-1	1911167	1954919	*Rhizobium sp.*	Other clade in Rhizobiaceae	17	CP088010[e]
CP002447-1	3975269	3937865	*Mesorhizobium cicerci*	Clade in Phyllobacteriaceae	40	CP015064[f]
BA000012-1	347766	310089	*Mesorhizobium loti*	Clade in Phyllobacteriaceae	40	CP016079

[a]The name is the nucleotide accession number of the bacterial chromosome with a suffix to distinguish among more than one prophage in the same chromosome.

[b]Coordinates include left and right core attachment sites (AttL, AttR), defined as a direct repeat surrounding the prophage but appearing only once in an uninserted homologous chromosome. All of these Att sites are derived by insertion within a tRNA gene. AttR, by convention, is the site downstream in the orientation of the structure gene module.

[c]This and other uninserted chromosomes have a fragmented end of an integrase from yet another prophage remnant downstream of the tRNA-Pro used by CP021793-1 as an attachment site.

[d]There is a tRNA-Lys where attL is expected, but a repeat of it is not detected where attR should appear. All three "f" clade prophage candidates have a syntenic right flank. None of the "f" clade phages have an identifiable integrase, and they may have additional regions deleted. Coordinates listed include at least one flanking gene on each end. However, the history relating phages in this clade appears to have been mostly under selective pressure for function. (see table S2B).

[e]CP088010 appears to have the same attL and attR duplication around a 24 bp insert.

[f]In addition to the GT1-like prophage insertion relative to CP015064, CP002447 has a fragment of an additional prophage inserted in the tRNA-Ser forming the CP002447-1 AttL site and extending leftwards that CP015064 does not have either.

Table S2B. Replacement/synomymous analysis of selective pressure in the Sino "m" clade and the Sino "f" clade.

Compare		% AA ID	R	S	FrR	FrS	FrR/FrS
Sino "m"<br>CP019488 vs. CP021818-2							
	lg. terminase	88	66	142	0.066	0.44	0.15
	portal	97	34.5	110	0.022	0.23	0.09
	tubeB	95	37	116	0.031	0.28	0.11
Sino "f"<br>CP013051 vs. CP024307							
	lg. terminase	99	5.5	39.5	0.0053	0.12	0.046
	portal	97	28.2	53.8	0.018	0.11	0.17
	tubeB	95	32	95	0.027	0.23	0.12
Average							
	Sino "m"						0.12
	Sino "f"						0.11

Computed as in methods section 2.11.

Table S3. Mass spectrometric detection of proteins in phiGT1 virions.

Protein	Function	MW (kD)	SC[a]	SC/MW	Expected copy number
gp8	MCP	36	178	4.9	420
gp17	IVPB/gene7	36	45	1.3	12
gp18	IVPC/gene16	69	77	1.1	12
gp19	IVPD/gene20	54	41	0.76	30
gp3	portal	79	53	0.67	12
gp21	tail fiber	31	20	0.64	18
gp14	tubeA$_{Pr}$	22	13	0.59	3
gp22	tail fiber/ antireceptor	90	46	0.51	18
gp13	tubeA$_L$	23	9	0.39	3
gp11	head module	12	4	0.33	
gp5	head module	41	12	0.29	
gp46	nonstructure module[b]	11	2	0.18	
gp4	head module	43	7	0.16	
gp34	nonstructure module[b]	20	3	0.15	
gp15	tubeB	55	8	0.15	6
gp7	scaffold	34	2	0.059	<1

[a]SC - Count of spectra obtained at 95% confidence.
[b]Contamination with other host protein complexes such as ribosomes, elongation factors, and chaperonins, is also present at SC/MW < 0.2, and may explain the presence of the two proteins from nonstructure modules.

References:

Battistuzzi, F.U., Feijao, A., Hedges, S.B., 2004. A genomic timescale of prokaryote evolution: insights into the origin of methanogenesis, phototrophy, and the colonization of land. BMC Evol. Biol. 4, 44. doi:10.1186/1471-2148-4-44.

Cermakian N, Idekda TM, Miramontes P, Lang BF, Gray MW, Cedergren R. 1997. On the evolution of the single-subunit RNA polymerases. J. Mol. Evol. 45:671-681.

Ceyssens P-J, Hertveldt K, Ackermann H-W, Noben J-P, Demeke M, Volckaert G, Lavigne R. 2008. The intron-containing genome of the lytic Pseudomonas phage LUZ24 resembles the temperate phage PaP3. Virology 377: 233-238. doi: 10.1016/j.virol.2008.04.038.

Hardies SC, Thomas JA, Black L, Weintraub ST, Hwang CY, Cho BC. 2016. Identification of structural and morphogenesis genes of *Pseudoalteromonas* phage ϕRIO-1 and placement within the evolutionary history of Podoviridae. Virology 489: 116-127. doi: 10.1016/j.virol.2015.12.005.

Johnson MC, Sena-Velez, M, Washburn BK, Platt GN, Lu S, Brewer TE, Lynn JS, Stroupe ME, Jones KM. 2017. Structure, proteome and genome of Sinorhizobium meliloti phage FM5: A virus with LUZ24-like morphology and a highly mosaic genome. J. Str. Biol. 200:343-359. DOI: 10.1016/j.jsb.2017.08.005.

Serwer P, Hayes SJ, Zaman S, Lieman K, Rolando M, Hardies SC. 2004. Improved isolation of undersampled bacteriophages: finding of distant terminase genes. Virology 329:412-424. doi: 10.1016/j.virol.2004.08.021.

Wang S, Luo H. 2021. Dating Alphaproteobacteria evolution with eukaryotic fossils. Nat. Commun. 12:3324. doi:10.1038/s41467-021-23645-4.
